# Flagellar regulation in enterotoxigenic *E. coli* modulates innate immune activation

**DOI:** 10.1128/iai.00336-26

**Published:** 2026-09-17

**Authors:** Pauline Ikpa, Amanda Nengel, James B. Kaper, Mark Donowitz, Jennifer Foulke-Abel

**Affiliations:** 1 Division of Gastroenterology and Hepatology, Department of Medicine, Johns Hopkins University School of Medicine, Baltimore, Maryland, USA; 2 Department of Microbiology & Immunology, University of Maryland School of Medicine, Baltimore, Maryland, USA

**Keywords:** intestinal organoid monolayers, primary monocyte-derived macrophages, IL-17C, CXCL6, vaccine strain engineering

## Abstract

*Enterotoxigenic Escherichia coli* (ETEC) remains a leading cause of diarrheal disease in low- and middle-income countries, yet vaccine efforts have struggled to achieve broad and durable protection. To better understand early steps in host-pathogen interactions, we utilized human stem cell-derived jejunal organoid monolayers to compare epithelial responses to ETEC H10407 and E24377A, prototypical strains frequently used in controlled human infection challenges. Transcriptomic analysis revealed that H10407 uniquely stimulated a robust innate immune response, characterized by upregulation of TLR5-dependent genes, including *IL-17C* and *CXCL6*. This response was found to be driven by sustained expression of flagella, the result of a naturally occurring *fliZ* gene disruption in H10407, which allows flagellin production despite ample nutrient availability. Complementation with *fliZ* restored repression of flagellar genes, confirming its regulatory role. In epithelial-macrophage co-cultures, H10407-expressed flagella enhanced macrophage sampling of bacteria as well as IL-6 production, effects that were not replicated by recombinant flagellin alone. However, exogenous flagellin did induce epithelial IL-17C release, a cytokine implicated in adaptive immune priming. These findings identify constitutive flagellar expression as a key factor in the heightened innate immune response and virulence of H10407, and highlight its potential utility in optimizing mucosal vaccine design against ETEC.

Enterotoxigenic *Escherichia coli* (ETEC) is a major diarrheal pathogen endemic in low- and middle-income countries, contributing to repeated illness, developmental delays in young children, and preventable deaths (1). Vaccine development efforts have not yielded durable efficacy against infection, perhaps in part due to the range of non-conserved surface antigens present among ETEC strains (2, 3).

Gaps persist in understanding how bacterial pathogenic factors are regulated in the human host environment, including which factors are critical for stimulating innate immune recognition, and how innate signals ultimately translate to adaptive immunity. Characterization of early steps in sensing of ETEC strains by human intestinal mucosa could provide important insight into not only understanding the bacterial signals that impart disease severity but also informing antigen inclusion for vaccine engineering.

In this study, we used normal human adult stem cell-derived jejunal organoid monolayers to characterize the epithelial response to ETEC. We detected increased inflammatory molecules induced by altered flagellar regulation in H10407 relative to other ETEC strains (E24377A or B7A). Organoid monolayers were then co-cultured with primary monocyte-derived macrophages to interrogate the importance of bacterial flagella in promoting immune cell sampling/phagocytosis across the epithelial barrier. Our observations support the importance of flagellar expression in facilitating mucosal recognition and response to ETEC infection.

## RESULTS

### ETEC H10407 induces epithelial responses that are distinct from ETEC E24377A

We used the prototypical ETEC strains H10407 and E24377A to profile differences in epithelial response to infection. Both strains produce heat-labile enterotoxin (LT), heat-stable enterotoxins (ST_h_ and ST_p_), and the flagellar tip adhesin EtpA, but differ by expression of colonization factor CFA/I in H10407, and CS1 and CS3 in E24377A. Jejunal organoid lines established from four different human donors were plated as monolayers on permeable supports, differentiated to form a mature epithelial barrier, and then infected (1 × 10^7^ CFU/mL inoculum, 6 h) to probe the intestinal epithelial response by bulk RNA-seq. Principal component analysis (PCA) indicated that donor identity was the primary source of transcriptional differences in the organoids, with infection status being a secondary factor (Fig. 1A). Interestingly, a larger number of significant differentially expressed genes (DEGs, ∣log_2_FC∣ ≥ 1, *P* ≤ 0.05) were associated with exposure to H10407 (307 increased, 256 decreased) (Fig. 1B; Table S1) than E24377A (58 increased, 154 decreased) (Fig. 1C; Table S2). Comparison of DEGs between H10407 and E24377A revealed 146 transcript changes that were shared, 417 DEGs unique to H10407, and 66 DEGs specific to E24377A (Fig. 1D; Table S3). Gene Ontology biological process terms associated with the upregulated DEGs common to both strains were weakly indicative of inflammatory signaling (FDR = 1.69e-2). The upregulated DEGs specific to H10407 infection included many additional chemokines and genes associated with inflammation/NF-kB activation (FDR = 2.68e-11) (Fig. 1E).

The top transcripts that were induced equivalently by both strains included immune components *C2CD4A*, *IL20*, *CXCR4*, *CSF2*, *CXCL2*, *IL1A*, and *CX3CL1*. The presence of hypoxia targets *HILPDA*, *DDIT4*, and *VEGFA* was suggestive of bacterial oxygen consumption near the epithelial surface (Fig. 1F). DEGs specific to H10407 infection included *OLR1*, the lectin-like oxidized low-density lipoprotein receptor 1 (LOX-1), and *TNFAIP6*, which encodes tumor necrosis factor-stimulated gene 6 (TSG-6). OLR1/LOX-1 has been described to play a role in host-pathogen interactions via prominent expression in macrophages, dendritic cells, and neutrophils, as well as inducible expression in lung and gastric mucosa (4-6). TNFAIP6/TSG-6 is a secreted tissue-protective factor that serves to limit excessive inflammation. Numerous cytokine and chemokine transcripts including *CCL5*/*7*/*8*/*20*, *IL17C*, *IL19*, *CXCL6*, and *IL1B* were also uniquely increased by H10407 relative to E24377A (Fig. 1G). The results suggested that H10407 infection, which can present as more clinically severe than E24377A, causes increased epithelial inflammatory signaling that is independent of professional immune cell participation.

### Epithelial responses unique to strain H10407 are caused by flagellin

Alongside ETEC H10407 and E24377A, we followed up with testing exposure to ETEC B7A and nonpathogenic *E. coli* HS for their potential to induce equivocal innate immune responses in organoid monolayers. All of the strains tested contain lipopolysaccharide (LPS) in their outer membranes and are able to express flagella, so these purified ligands were tested in parallel with live bacterial exposures in organoid monolayers and relative changes in *IL17C* and *CXCL6* levels were measured by qRT-PCR (Fig. 2A and B). Similar to E24377A, neither B7A nor HS substantially induced *IL17C* or *CXCL6* transcripts in the monolayers. LPS O55:B5, a validated TLR4 ligand, also did not mimic the effects of H10407. However, purified FliC from *S. typhimurium*, a ligand of TLR5, was sufficient to significantly upregulate *IL17C* and *CXCL6*, suggesting that epithelia were responding to the presence of flagella on H10407.

We next used a flagella-deficient H10407 derivative (Δ*fliC*)(7) to directly test the role of flagella in epithelial stimulation. Organoid monolayers with attenuated TLR5 expression (stable knockdown with shRNA, TLR5 KD) were also generated to verify the receptor requirement, and CXCL6 secretion was employed as a readout of activation (Fig. 2C). H10407 Δ*fliC* was unable to induce CXCL6 secretion in organoid monolayers, and TLR5 KD significantly limited stimulation by wild-type (WT) H10407 (Fig. 2D). We further employed H10407 Δ*fliC* to confirm that several other major innate immune response targets identified by RNA-seq were similarly dependent on flagellar expression (Fig. 2E). Together, these experiments verified that flagella were required to elicit genes with potential significance in conveying pathogenic presence to professional immune cells (*CCL8*, *CD83*, *CXCL6*, and *IL17C*) or preparation for infection defense (*OLR1*, *PGLYRP2*).

### Differential induction of epithelial TLR5 is due to sustained flagellar expression by H10407

To understand the factor(s) in H10407 contributing to unique activation of TLR5, we began by testing the ability of heat-killed (HK) strains to induce a TLR5/NF-κB reporter engineered in HEK293 cells (HEK-Blue/hTLR5) (Fig. 3A). HK-H10407 activated the TLR5/NF-κB reporter, but the other HK strains (B7A, E24377A, HS) failed to elicit a response. HK-TOP10 *E. coli*, which is an aflagellate strain, was included as a negative control. This confirmed the observations made in organoid monolayers regarding specific activity toward H10407 but did not resolve the origin of differential TLR5 activation.

We next considered that the distinctive epithelial response to H10407 could be driven by a variable region in FliC that would alter TLR5 binding or activation relative to the other strains. To test this hypothesis, recombinant N-terminal His-tagged proteins (rFliC) from H10407 (H11), ETEC B7A and E24377A (H28), and nonpathogenic HS (H4) were expressed and purified for incubation with HEK-Blue/hTLR5 cells (Fig. 3B). The possibility of NF-κB activation in the absence of TLR5 involvement was also assessed in a separate reporter cell line (HEK-Blue/Null1). Additional controls included recombinant FliC from *S. typhimurium* (rFla STm) (TLR5-specific ligand), LPS O55:B5 (TLR4-specific ligand), and TNFα (TLR-independent NF-κB activator). In contrast to the HK strain experiment in Fig. 3A, all three rFliC proteins were robust TLR5-dependent activators of the reporter (Fig. 3C). As a final attempt to discriminate between H11 and H4/H28, the HEK-Blue/hTLR5 reporter assay was conducted using a range of rFliC concentrations to detect potential differences in concentration-based affinity for TLR5. However, the assay indicated that the rFliC proteins had indistinguishable activity with calculated EC_50_ values in the low nM range (EC_50_ = 1 nM for H11, 3 nM for H4 and H28) (Fig. 3D).

In a further attempt to resolve the discrepancy between HK strains and their respective rFliC proteins observed in TLR5 reporter assays, we postulated that culture media composition could be affecting flagellar gene regulation. In *E. coli*, the flagellar biosynthetic operon is regulated by carbon source availability. For infection experiments with organoids, bacterial cultures were routinely expanded under aeration in tissue culture medium containing D-glucose (0.3% wt/vol) to maintain compatibility with human cell culture. To test for strain differences in carbon catabolite repression of the flagellar operon, *fliC* expression levels were compared in bacterial cultures obtained from D-glucose-containing medium or standard microbiological medium (glucose-free), which provides oligopeptides as an energy source (Fig. 3E). As expected, D-glucose significantly downregulated *fliC* as well as *flhD,* a master regulator of the flagellar operon, in all strains relative to the glucose-free condition. However, H10407 sustained a level of *fliC* expression in the presence of D-glucose that was approximately 10-fold greater than B7A or E24377A under the same conditions. This suggested that H10407 harbors a mechanism to partially resist carbon catabolite repression (CCR) of flagellar biosynthesis. Evaluation of the flagellar operon master regulator *flhD* by qRT-PCR provided similar results, with D-glucose imposing downregulation relative to glucose-free medium in all strains, although the effect was significantly less pronounced in H10407 than in B7A or E24377A (Fig. 3E).

### Functional loss of the global repressor FliZ in H10407 permits constitutive flagellar expression

Flagellar biosynthesis is a complex network of processes regulated at multiple levels. The master motility regulator FlhDC facilitates the switch between motile and sedentary states and is itself regulated by several transcription factors. Among the regulators of *flhDC* is FliZ, previously shown to antagonize the binding of stress factor σ^S^ to the *flhDC* promoter (8). Review of the published H10407 genome (9) indicated that the *fliZ* sequence was disrupted by *insB*, a part of the transposable element IS1. This insertion introduced a premature stop codon that would prevent expression of full-length FliZ, potentially altering regulation of flagella biosynthesis. We confirmed the *fliZ* gene disruption in H10407 by comparison with *E. coli* K-12 genomic DNA via PCR and Sanger sequencing (Fig. 4A). Sequence alignments also revealed that the *flhDC* promoter region (flhDC*p*) in H10407 has two mutations and a base pair insertion near the predicted binding site for multiple regulators that include FliZ (Fig. 4B). Together, these observations prompted investigation of the potential role played by absent FliZ and/or *flhDC* promoter mutations in dysregulated flagellar expression in H10407.

An electrophoretic mobility shift assay (EMSA) was used to evaluate binding of full-length recombinant FliZ (rFliZ) (Fig. 4C) to *flhDC* promoter fragments from K-12 or H10407. Similar band shifts were observed using the respective promoter sequences at equivalent concentrations of rFliZ (Fig. 4D). This result suggested that if *fliZ* were intact in H10407, the mutations in the *flhDC* promoter were not likely to contribute to aberrant motility regulation.

We next tested for the ability of complemented *fliZ* to demonstrate native-like repression of FliC expression in H10407. Since the expression construct used in the EMSA experiment had produced rFliZ with functional *flhDC* promoter binding ability, the same plasmid was introduced into H10407. IPTG induction of FliZ expression effectively lowered FliC expression in H10407, demonstrating that the mutational inactivation of *fliZ* allowed flagellar production (Fig. 4E). Because *flhD* and *fliC* were not completely inhibited by D-glucose availability in H10407 (Fig. 3E), this result also suggested that FliZ may impose flagellar regulation that is independent of the CCR pathway.

### Flagellar expression promotes sampling of H10407 by macrophages co-cultured with epithelia

With an understanding of the importance of flagella in stimulating epithelial innate immune responses to ETEC, we next questioned how these signals would affect the way that professional immune cells such as tissue-resident macrophages behave during infection. To model these interactions, organoid monolayers were coupled with resting monocyte-derived macrophages in co-cultures as previously described (10). Permeable tissue culture support membranes with 3 μm pores were used to permit bacterial transfer from the upper epithelial surface to macrophages plated on the bottom side of the membrane (Fig. 5A). Infection increased the average transepithelial electrical resistance (TER) measured in both monolayers and co-cultures after 6 h infection, and this effect was independent of strain identity (Fig. 5B). Thus, bacteria that appeared in the basolateral chamber associated with macrophages were likely the result of active immune cell sampling rather than injury-associated epithelial breaches. Phagocytosis of bacteria by macrophages across the organoid monolayer was visualized and enumerated by immunostaining and confocal microscopy (Fig. 5C). Co-cultures infected with H10407 Δ*fliC* yielded significantly fewer phagocytosed bacteria per macrophage than that observed with WT H10407 (Fig. 5D), suggesting that the signaling afforded by flagella stimulated macrophage sampling of the epithelial surface. In a separate set of co-culture experiments, infection with HS (carrying an mScarlet fluorescent reporter) elicited minimal macrophage sampling and phagocytosis (Fig. 5E and F). However, when a mixture of HS and H10407 was added to co-cultures, macrophages sampled HS more frequently, implying that flagella-activated signaling could increase sampling frequency.

We next conducted assays in macrophages alone to establish responsiveness to infection-associated signals. A migration assay was conducted to evaluate macrophage mobilization by epithelial-specific products IL-17C and CXCL6 stimulated by H10407. Monocyte-derived macrophages were plated in the top chamber of an 8-μm pore membrane, and the recombinant chemoattractant was introduced to the bottom chamber to stimulate cell movement across the membrane. A migration index for each signal was calculated relative to the random migration of macrophages in an untreated chamber. Both rIL-17C and rCXCL6 initiated an approximately 2-fold increase in migration, suggesting that these epithelial signals may contribute to the increased sampling of H10407 (Fig. 5G). As a readout of macrophage responsiveness to microbial-associated molecular patterns (MAMPs), *IL-6* transcripts were measured in response to WT H10407, the Δ*fliC* mutant, or purified rFliC. Each strain, as well as rFliC alone, was sufficient to increase *IL-6* mRNA in macrophages upon direct exposure (Fig. 5H).

Our final objective was to determine if rFliC could be employed to stimulate macrophage engagement with bacteria that normally downregulate flagella within the host environment and thus appear to be sampled at a lower frequency than H10407. First, we confirmed that *IL-17C* was primarily induced in epithelia and that *IL-6* was predominantly stimulated in macrophages by conducting direct infection of each monoculture with H10407 and evaluating transcripts by qRT-PCR (Fig. 6A). Co-cultures were then infected with or without rFliC-H11, and the basolateral medium was tested for IL-17C content to establish that epithelia were activated at similar levels by H10407 and by H10407 Δ*fliC* or E24377A supplemented with rFliC. After establishing a dose of rFliC that achieved IL-17C induction similar to the effect of H10407, macrophage-specific production of IL-6 was measured in these samples to determine the relative sampling frequency for each strain supplemented with rFliC. While rFliC appeared to provide competent epithelial activation, indicated by IL-17C production (Fig. 6B), it was not sufficient to promote bacterial phagocytosis by macrophages, as measured by IL-6 release (Fig. 6C). Together, these results suggest that administering exogenous rFliC does not replace the function of natively expressed flagella in the context of mucosal macrophage sampling and IL-6 release. However, rFliC appears to be sufficient to attain epithelial production of IL-17C, which could participate in adaptive immune responses for purposes of vaccine administration.

## DISCUSSION

Three of the most commonly used ETEC strains to study immune responses in controlled human infection models (CHIMs) are H10407, B7A, and E24377A (11). Naïve human volunteers exposed to H10407 routinely experience larger diarrheal volumes for a longer duration than those infected with either B7A or E24377A, but the origins of these virulence disparities are not entirely clear (11). Interestingly, CHIMs have also found that H10407 exposure provides strong protection upon rechallenge (12), suggesting that the features conferring virulence in H10407 may also be critical antigens for vaccine design. Many efforts have relied on the immunogenicity of LT, yet the live attenuated vaccine candidate ACE527 adjuvanted with double mutant LT (dmLT) had somewhat limited efficacy (13). A review of the three strains in ACE527 and their immune responses revealed unintended absence of factors including the immunogenic flagellar tip adhesin EtpA and flagellar expression, causing loss of motility (14). The current study findings support that restoration of flagella may be necessary to improve vaccine performance.

To investigate potential differences in mucosal responses to ETEC strains, we began by comparing the transcriptomes of intestinal organoid monolayers infected with H10407 or E24377A and found that although the strains share expression of several virulence factors (LT, ST_h_, ST_p_, EtpA), there were many DEGs that centered on an innate immune response specific to H10407. The upregulated transcripts were consistent with TLR activation, and through comparisons with additional strains and purified bacterial components, it was determined that the likely source of this distinction was related to flagella.

The *E. coli* flagellar filament protein FliC is detected by intestinal epithelial cells via TLR5. Across bacterial phyla, FliC homologs have core conserved regions but can vary significantly in molecular weight and within hypervariable regions, some of which allow pathogens to evade detection by TLR5 (15). Recent studies have described a spectrum of TLR5 activation potential exhibited by commensal and pathogenic microbes. Additionally, variations in FliC glycosylation were also reported in a mass spectrometry-guided comparison of H10407 and *E. coli* K-12 (16). Given these prior investigations, we initially hypothesized that H10407 may have domains or post-translational modifications in FliC that would explain the differential effect we observed in epithelia. However, we found that purified rFliC from the respective strains demonstrated equivalent ability to activate a TLR5 reporter. This suggested that potential differences in H10407 gene regulation could account for the unique epithelial innate immune stimulation we observed through RNA-seq.

This study demonstrated that the frameshifting inactivation of *fliZ* in H10407 confers a low level of constitutive flagellin expression in the presence of an energy source (glucose), which is a condition that normally represses motility genes in *E. coli* to conserve energy. The *insB::fliZ* insertion in H10407 is documented in public genomic databases (9) and was confirmed in our samples. This mutation allows H10407 to promote epithelial responses via TLR5 activation, including release of the cytokine IL-17C, that are not robustly stimulated by other ETECs (B7A, E24377A, H10407 Δ*fliC*) or nonpathogenic *E. coli* strains (HS) under repressive conditions. FliZ is especially abundant in the post-exponential growth phase of *E. coli* (8) and has been shown to exert feedback control of flagellar gene expression through binding upstream of *flhDC*, effectively blocking the inhibitor σ^S^ from binding (8). Because FliZ binds to DNA sequences that overlap with the −10 region of σ^S^-dependent promoters, it functions as a global repressor of numerous genes unrelated to motility (8, 17). Thus, there may be other effects on virulence characteristics in H10407 affected by FliZ loss that were peripheral to the scope of this study.

We observed that plasmid-borne complementation of a WT *fliZ* decreased FliC expression in H10407, confirming that functional loss of FliZ was allowing a level of sustained flagellar expression. H10407 was previously observed to downregulate flagella following successful adherence to Caco-2 intestinal epithelial cells (18), but prior to this interaction, flagellum-mediated motility is critical to penetrate the intestinal mucus barrier and make initial contact with the epithelial surface (7). Continuous flagellar production occurs in pathogenic bacteria such as *Vibrio parahaemolyticus* and *Aeromonas hydrophila*, but the operons are regulated quite differently than in *E. coli* (19, 20). In adherent-invasive *E. coli* (AIEC), flagellin was noted to be upregulated by the host intestinal environment and contributed to pathogenicity (21). Genetic knockouts that abolish flagella in these strains significantly reduce biofilm formation and colonization (19-21). Since ETEC flagella impart motility as well as bacterial adhesion to epithelia, and both functions are known to be important for efficient delivery of heat-labile toxin (LT) to host cells (7, 22, 23), this could in part explain the enhanced virulence noted for H10407 infection relative to other strains (11).

Flagellar expression in H10407 also increased the frequency of bacterial sampling/phagocytosis and yielded release of IL-6 by macrophages in co-culture with infected epithelia. Surprisingly, exogenous application of rFliC was insufficient to promote macrophage sampling of flagella-deficient strains (E24377A, H10407 Δ*fliC*), suggesting that the adherence and/or motility functions of flagella may be important to facilitate antigen sampling of bacteria across the epithelial barrier. This ambiguity could be addressed in the future by generating flagellar motor mutants in H10407 that express the fully assembled flagellar filament but are paralyzed and cannot actively swim.

In contrast to the effect on macrophages, addition of rFliC was able to elicit IL-17C release in epithelia colonized with flagella-deficient strains. IL-17C is upregulated in human colonic epithelial cell lines following exposure to flagellin (24, 25), gastric mucosa colonized by *H. pylori* (26), and mice infected with *C. rodentium* (27). It appears to be important for host defense against microbes, since *Il17c*−/− and *Il17re*−/− mice exhibit severely impaired epithelial recovery from both pathogen exposure and the microflora-induced inflammation of DSS colitis (25, 27). IL-17C was first described to bind and activate monocytic THP-1 cells (28). While we found that rIL-17C was a moderate stimulus for macrophage migration in a chamber assay, it did not appear to promote engagement of antigen-presenting cells with bacteria in epithelial co-cultures. More recently, IL-17C has been recognized as a regulator of Th17 cell differentiation, enhancing the production of IL-17A and IL-17F (29). Th17 cells can promote IgA secretion and differentiate into long-lived tissue-resident memory T cells, so flagella could support this aspect of mucosal immune development to maximize ETEC live attenuated vaccine responses.

In summary, our study found that H10407 maintains a level of constitutive flagellar expression due to genetic inactivation of the regulator fliZ. While this phenotype incurs some energetic cost, it also seems to support bacterial survival, colonization, and innate immune activation within the host GI tract. By demonstrating increased epithelial signaling and macrophage engagement/sampling of H10407 relative to ETEC E24377A, ETEC B7A, or nonpathogenic *E. coli* strain HS, this study contributes mechanistic insight regarding the pathogenicity of H10407 infection and the associated innate immune response. Furthermore, considering that H10407 clinically presents in CHIMs with prolonged virulence and increased protective efficacy upon homologous rechallenge when compared to E24377A or B7A, this work sheds additional light on bacterial genotypic and phenotypic characterizations that may underlie these observations and supports flagellar expression as an important consideration for vaccine antigen design.

## MATERIALS AND METHODS

### *E. coli* strains

Enterotoxigenic *E. coli* strain H10407 and the Δ*fliC* mutant were kind gifts from James Fleckenstein, Washington University School of Medicine. Enterotoxigenic *E. coli* strains B7A and E24377A were provided by David Sack, Johns Hopkins Bloomberg School of Public Health. *E. coli* HS is a nonpathogenic strain from existing laboratory stocks at the University of Maryland. A complete list of strains is available in Table S4.

### Plasmid cloning

Custom GeneArt constructs in plasmid pMK (ThermoFisher) for fliC-H4, fliC-H11, and fliC-H28 were designed with codon-optimized sequences to eliminate native restriction enzyme recognition sites to be used in subsequent cloning. The *fliC* gene for each strain of interest (H4, *E. coli* HS; H11, H10407; H28, B7A/E24377A) was excised from the respective pMK-fliC plasmid using *BamH*I and *Hind*III, gel purified, and ligated to pTrcHis A. The *fliZ* gene was amplified from *E. coli* K-12 genomic DNA using primers flanked by restriction enzyme sites *BamH*I and *EcoR*I. DNA fragments were gel purified, digested, and ligated to linearized pTrcHis A plasmid with T4 DNA ligase to generate N-terminal 6× His-tagged fusion proteins. Chemically competent NEB-5α cells were transformed with the ligation mixture and plated on LB agar with 100 μg/mL ampicillin selection to obtain clones. Several colonies from each transformation were miniprepped and verified by Sanger sequencing. To generate the mScarlet-I fluorescent reporter plasmid, the gene was PCR-amplified using primers flanked by *KpnI* and *HindIII*, gel purified, and ligated to pUC19. The plasmid was subsequently introduced to chemically competent *E. coli* HS to generate the reporter strain under ampicillin selection. A complete list of strains and plasmids is available in Table S4, and primer sequences are listed in Table S5.

### Organoid culture

Human intestinal organoids from a preexisting biobank (Table S6) were used in all experiments described. These jejunal lines were established from endoscopic biopsies or surgical resections following informed consent under institutional review board approval at Johns Hopkins Hospital (NA_00038329, IRB00106944, or IRB00367478). Organoid propagation in Matrigel and as monolayers on permeable cell culture inserts has been described previously (10, 30, 31). Briefly, organoids were expanded as Matrigel-embedded cultures in expansion medium (EM) containing Advanced DMEM/F-12, 10 mM HEPES, 1× GlutaMax, 1× B-27 supplement, 50% (vol/vol) Wnt3A conditioned media, 15% (vol/vol) R-spondin one conditioned media, 10% Noggin conditioned medium, EGF, A83-01, SB202190, and 1× Primocin. Cultures were fed every 2–3 days and split every 7 d. Monolayers were plated on inserts (Corning) coated with collagen IV (10 μg/cm^2^, Sigma) in NDM medium (EM without A83-01 or SB202190) until confluent and then differentiated for 5 days in DF medium (EM without Wnt3A, R-spondin 1, A83-01, SB202190, or Primocin) prior to use in all experiments.

### Primary monocyte-derived macrophages

Macrophages were obtained with minor modification of the procedure described by Noel et al. (10). Cryopreserved human peripheral blood mononuclear cells (PBMC, IQ Biosciences, Table S6) were thawed, enriched for monocytes using a Pan Monocyte Isolation Kit (Miltenyi Biotec), and seeded on 6-well plates at 10^6^ cells/mL in RPMI-1640 (Corning), 10% normal human serum (Sigma), 1× MEM non-essential amino acids, 1 mM Na pyruvate, 55 μM β-mercaptoethanol, 1× Primocin (Invivogen), and 50 ng/mL M-CSF (BioLegend). Complete medium was changed on day 3, and cultures were refed every 3–4 days thereafter without additional M-CSF. Monocyte-derived macrophages were used on day 6 or day 13 for direct infection or co-culture experiments as indicated in figure legends.

### Direct macrophage infection

Macrophages (10^5^ cells/well) seeded in a 6-well plate were exposed to bacteria (MOI 100) or rFliC (50 μM) at 37°C, 5% CO_2_ atmosphere for 4 h. Wells were washed three times with excess PBS prior to harvesting macrophage mRNA.

### Organoid monolayer-macrophage co-culture

Macrophages were dislodged from culture plates with Accutase and plated onto the bottom of inverted 3.0-μm-pore inserts containing partially differentiated organoid monolayers as described by Noel et al. (10). Newly seeded co-cultures were allowed to rest in DF medium for 24 h prior to infection.

### Monolayer and co-culture infection

Bacterial strains were grown overnight (~12–14 h) in Advanced DMEM/F-12, 37°C with aeration. All infections were conducted in antibiotic-free DF medium at 37°C, 5% CO_2_ atmosphere for 6 h, using 1 × 10^7^ CFU/mL inocula diluted from the overnight cultures.

### RNA sequencing and analysis

Differentiated organoid monolayers were infected with 1 × 10^7^ CFU/mL inocula in the upper chamber of the insert for 6 h at 37°C in a humidified 5% CO_2_ tissue culture incubator. Monolayers were washed three times with PBS, and total RNA was extracted with a PureLink RNA Micro Kit (ThermoFisher Scientific). Ribosomal RNA was depleted in the samples, and sequencing libraries were prepared using the NEBNext Ultra II Directional RNA Library Prep Kit for Illumina. Multiplexed libraries were sequenced on an Illumina NovaSeq X Plus at JH Genomics, Johns Hopkins University. Sequence data processing and analysis was completed using the Galaxy platform public server at usegalaxy.org (32-34). Each strain was compared to uninfected controls using DESeq2 to determine significant differentially expressed genes (∣log2FC∣ ≥ 1, *P* ≤ 0.05).

### Quantitative reverse-transcription PCR (qRT-PCR)

Transcripts were assayed by qRT-PCR. For organoid monolayers or cultured macrophages, total RNA was extracted using a PureLink RNA Micro Kit (ThermoFisher Scientific) and transcribed to cDNA with SuperScript VILO Master Mix (ThermoFisher Scientific). qRT-PCR was performed on a QuantStudio 12K Flex Real-Time PCR instrument (Applied Biosystems) using PowerUP SYBR Green Master Mix (Applied Biosystems) and oligonucleotide primers specific for the exon targeted by the shRNA (Table S5). Each sample was assayed in triplicate using 5 ng RNA-equivalent cDNA per reaction. Relative fold-changes in mRNA levels were calculated using 18S rRNA for internal normalization and the 2^−ΔΔC^_T_ method.

### TLR5 reporter assay

The HEK-Blue hTLR5 cell line (Invivogen) is transfected with the human TLR5 gene and a secreted alkaline phosphatase (SEAP) reporter gene under control of the IFN-β minimal promoter fused to five NF-κB and AP-1 binding sites. Stimulation with TLR5 ligands activates SEAP production. The HEK-Blue Null1 cell line (Invivogen), which carries the SEAP gene but lacks TLR5 expression, was used as a control in all experiments. The respective cell lines were exposed to heat-killed bacterial strains (10^8^ cells/mL), recombinant flagellins (50 μM), TNFα (100 ng/mL, positive control for SEAP induction), or LPS (100 ng/mL, negative control requiring TLR4 to activate SEAP) at 37°C, 5% CO_2_ for 16 h. Medium was collected to measure relative SEAP activity using QUANTI-Blue SEAP Detection Reagent (InvivoGen) absorbance in a microwell plate at 620 nm (AccuSkan plate reader, Fisher Scientific). To determine the EC_50_ for each recombinant flagellin, a range of protein concentrations (0.1, 0.25, 0.5, 0.75, 1, 2.5, 5, 7.5, 10, 100, and 1000 nM rFliC) was applied to HEK-Blue/hTLR5 cells for 16 h and analyzed as detailed above.

### TLR5 knockdown

Organoids in 3-D Matrigel culture were treated with 10 μM CHIR99021 for 2 days prior to transduction. Organoids were harvested from Matrigel and briefly digested (~3 min) with TrypLE (Thermo Fisher Scientific) at 37°C. Cells were washed with DMEM/F-12 medium, pelleted, and resuspended in EM containing polybrene (10 μg/mL) and lentivirus (≥10^6^ particles, GIPZ shRNA, GE Dharmacon) targeting sequences listed in Table S5. Cells were incubated at 32°C with shaking for 1 h, then transferred to a tissue culture incubator at 37°C, 5% CO2, for an additional 4 h. Cells were pelleted at 300 × g, plated in Matrigel, and fed with EM following standard culture methods. After 2 days, puromycin selection (5 μg/mL) was initiated and maintained throughout culturing. Relative knockdown efficiency was determined by qRT-PCR using primers specific to the TLR5 exon targeted by the shRNAs.

### Macrophage migration assay

Macrophages were dislodged from culture plates with Accutase. A suspension containing 1 × 10^5^ cells was plated on tissue culture inserts (8.0 μm pore, Greiner Bio-One) and allowed to attach/rest for 24 h. Treatments including rIL-17C, rCXCL6 (100 ng/uL or 1 μg/uL each), or vehicle control were added to the receiver plate wells and incubated at 37°C, 5% CO_2_ atmosphere for 24 h. Non-migrating cells that remained on the upper membrane surface were removed with a cotton-tipped swab. Inserts containing migrated cells on the bottom membrane surface were then washed with PBS, stained with Hoechst 33342, and imaged on an epifluorescence microscope with a CCD camera (Zeiss AxioObserver) for macrophage quantitation.

### Electrophoretic mobility shift assay

IRDye700-conjugated dsDNA fragments (250 bp) derived from the *flhDC* promoter sequence in H10407 or *E. coli* K-12 (a consensus wild-type promoter sequence) were prepared by PCR amplification with the labeled 20-mers (Integrated DNA Technologies), flhDCp-IRDye700 F and flhDCp-IRDye700 R (Table S2), and Q5 HiFi DNA polymerase master mix (NEB). The products were purified by agarose gel electrophoresis, isolated with a QIAquick Gel Extraction Kit (Qiagen), and quantified by nanospectrophotometry (DeNovix DS-11FX). Each reaction consisted of 100 ng IRDye700-flhDCp oligo, 0.05 μg poly dI·dC, 10 mM Tris pH 7.5, 50 mM KCl, 1 mM MgCl_2_, 1 mM EDTA, 2.5 mM DTT, 0.25% Tween-20, 5% glycerol, and varying concentrations of purified rFliZ protein (0, 0.5, 1.0, 2.5, or 5 μg). After 30-min incubation, binding reactions were separated on 6% TBE-PAGE at 100 V and visualized on a LI-COR Odyssey CLx infrared imager.

### Enzyme-linked immunosorbent assay

Basolateral medium was collected following 6 h infections as described above and stored frozen (−20°C) until use. ELISA plates were coated with capture antibody overnight, washed (PBS, 0.05% Tween-20), and then blocked for 1 h (PBS, 1% BSA). Standards and samples were assayed in duplicate following the manufacturer’s protocol for each kit (DuoSet ELISA for targets CXCL6, IL-17C, or IL-6, R&D Systems). Colorimetric detection was carried out using 1-Step Ultra TMB substrate (Thermo Fisher) followed by 2 N H_2_SO_4_ stop solution. Well absorbance was read at 450 nm with background correction at 620 nm (AccuSkan plate reader, Fisher Scientific).

### Immunostaining

Cells were washed with PBS, fixed with 4% paraformaldehyde/PBS, and incubated with antibodies against O78 (1:200, Abcam ab78826) and CD68 (1:50, BioLegend 333802) at 4°C overnight to detect H10407 and macrophages, respectively. The next day, cells were washed with PBS, incubated with donkey anti-rabbit IgG-Alexa Fluor 488, donkey anti-mouse IgG-Alexa Fluor 568, phalloidin-Alexa Fluor 647, and Hoechst 33342 (Thermo Fisher) for 1 h at 25°C. After washing with PBS, membranes were cut away from their polystyrene supports using a razor blade and mounted with ProLong Gold antifade on glass microscope slides for confocal imaging.

### Recombinant protein expression, purification, and immunoblotting

For expression and purification of His-tagged fusion proteins, LB Miller broth containing 200 μg/mL ampicillin was inoculated under aeration to 0.6 OD_600_ and then induced with 1 mM IPTG for 4 h. Cells were pelleted and stored frozen at −80°C until use. Thawed pellets were resuspended in TBS-I buffer (20 mM Tris pH 7.6, 150 mM NaCl, 10 mM imidazole) with protease inhibitors (cOmplete Protease Inhibitor Cocktail, Roche), sonicated, and centrifuged to pellet insoluble debris. The supernatant was mixed with Ni-NTA resin and rotated for 1 h at 4°C. The slurry was transferred to a plastic column, washed four times with TBS containing 20 mM imidazole, and eluted four times with TBS containing 500 mM imidazole. Eluates were combined and concentrated in an ultracentrifugation filter unit (10 kDa MWCO), diluted in PBS with 10% glycerol, aliquoted, and stored at −20°C.

For immunoblotting, bacterial pellets were lysed by bead milling in buffer (50 mM Tris pH 8.0, 100 mM NaCl, 1 mM EDTA) with protease inhibitors and centrifuged to pellet insoluble debris. The protein concentration of the supernatant was measured by Bradford assay (Protein Assay Dye, Bio-Rad). Lysates were separated by SDS-PAGE, transferred to a nitrocellulose membrane, and blocked with 3% BSA in TBS. Incubation with primary antibodies against 6×-His tag (1:2000, Invitrogen MA1-21315), flagellin/FliC (1:1000, Abcam ab93713), or DnaK (1:10,000, Enzo ADI-SPA-880-D) was conducted in 3% BSA, TBS, 0.1% Tween-20, with rocking overnight at 4°C. Washes and conjugated secondary antibody incubations (donkey anti-mouse IgG-Alexa Fluor 680 and donkey anti-rabbit IgG-Alexa Fluor 790, ThermoFisher) were carried out with rocking for 1 h at room temperature. Blots were scanned on a LiCor Odyssey CLx infrared imager, and band intensities were quantified in Image Studio (LiCor Biosciences).

### Statistical analysis

Statistical analysis was performed in GraphPad Prism 11. Student’s *t* test, one-way ANOVA, or two-way ANOVA with respective follow-up tests were applied to determine statistical significance (*P* < 0.05) as detailed in each respective figure legend.

## Supplementary Material

Supplementary Materials

**Supplemental Tables (IAI00336-26-s0001.docx).** Tables S1 to S6.

## Figures and Tables

**FIG 1:**
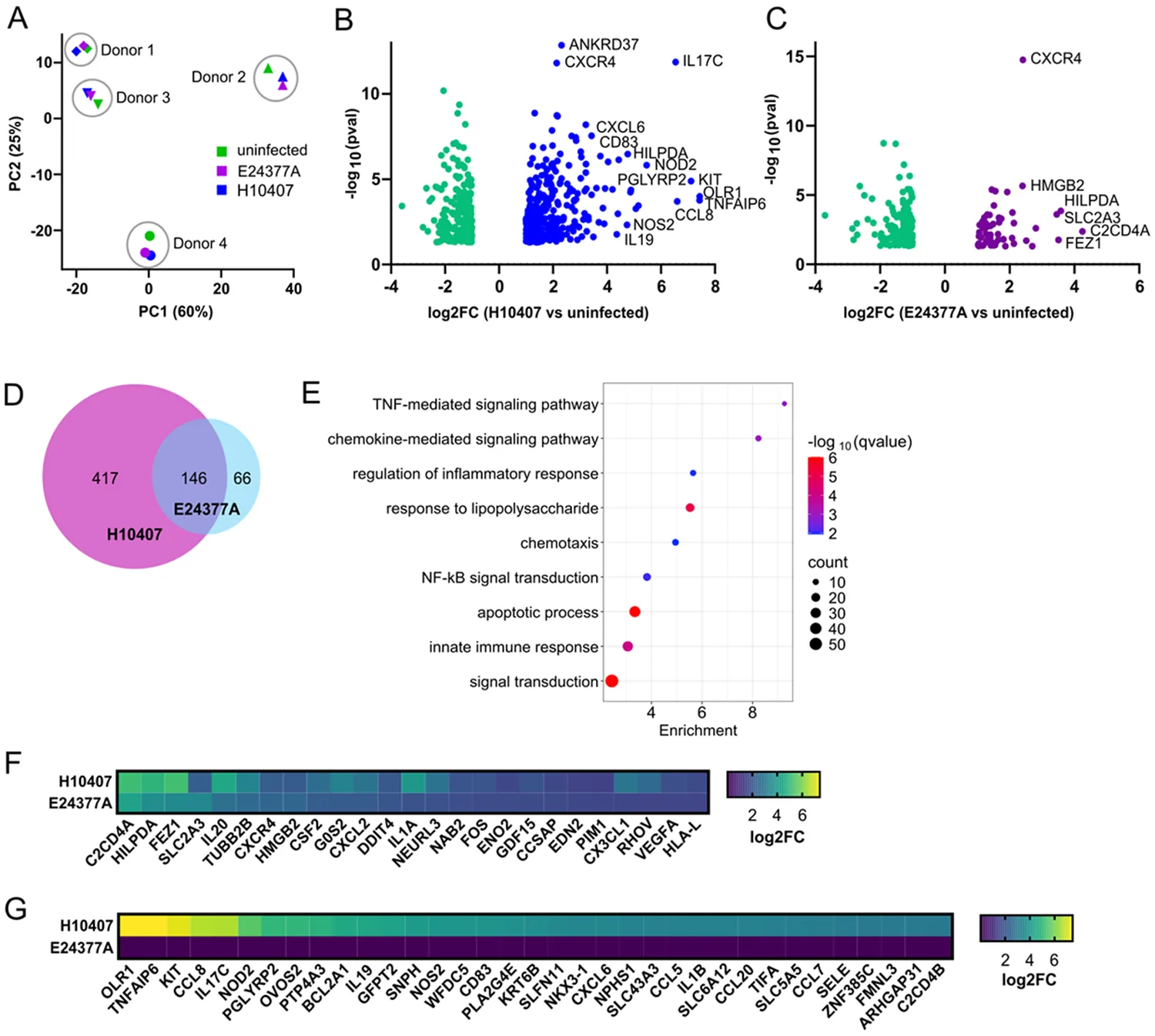
Epithelial transcriptome analysis following infection with ETEC H10407 or E24377A. Four unique donor organoid lines were exposed to H10407, E24377A, or vehicle (media) for 6 h prior to harvesting epithelial cells for RNA-seq. (A) Principal component plot for RNA-seq data comparing organoid donor and infection state. (B) Volcano plot of DEGs due to H10407 infection relative to uninfected epithelia. Downregulated genes are depicted in green, and upregulated genes are marked in blue. (C) Results of E24377A infection, similar to panel (B) Upregulated genes are marked in purple. (D) Venn diagram of upregulated DEGs to depict the number of common and pathogen-specific transcripts. (E) Fold enrichment of Gene Ontology Biological Processes upregulated by H10407 infection. (F) Heatmap of log_2_ fold changes in the top shared genes upregulated by both H10407 and E24377A infection relative to uninfected controls. (G) Heatmap of the top significant genes upregulated only by H10407 infection.

**FIG 2:**
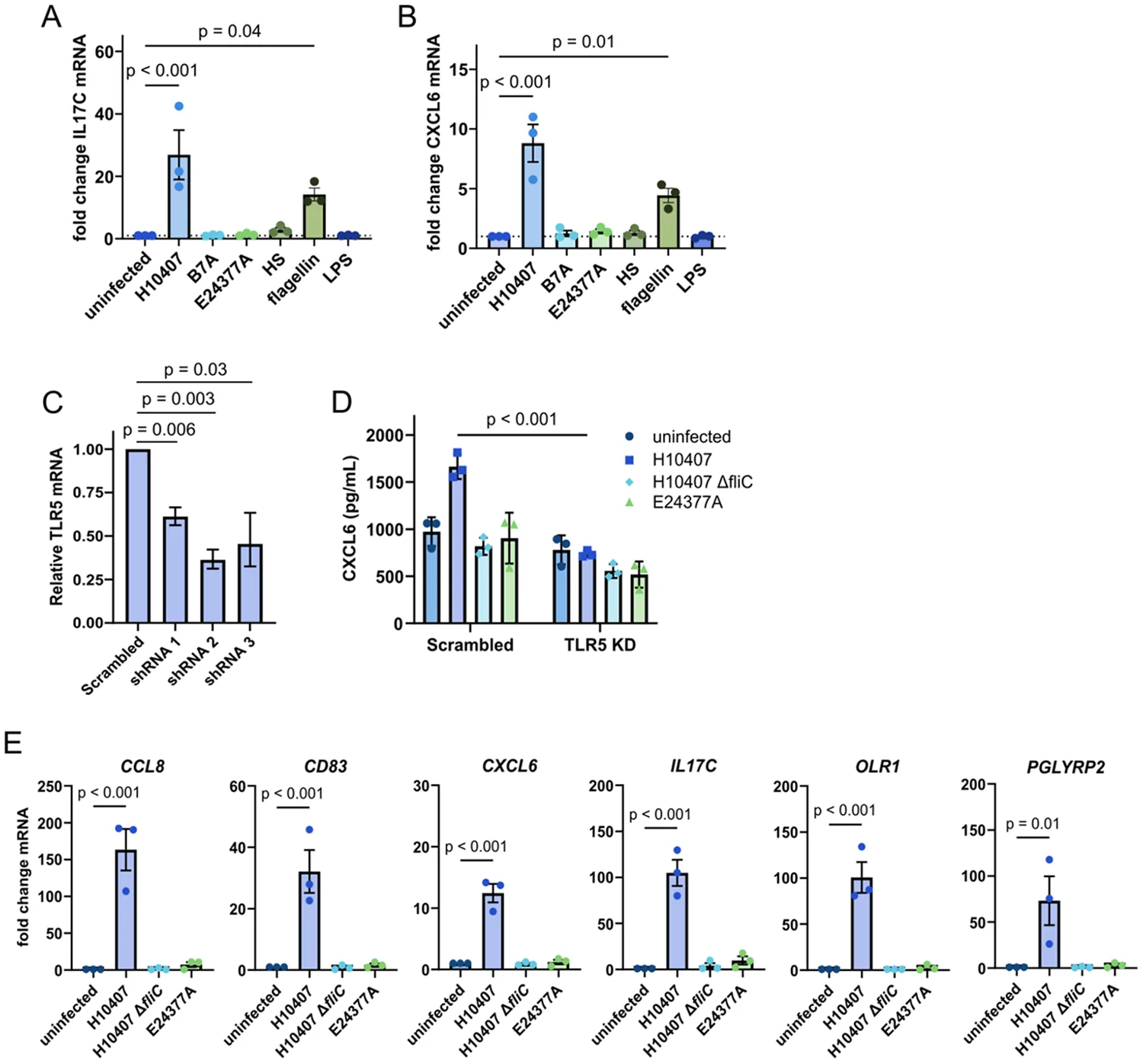
Epithelial innate immune responses to H10407 are caused by flagellin. (A) Fold change in *IL17C* transcripts induced by exposure to live bacteria or purified bacterial ligands. (B) Fold change in CXCL6 transcripts under conditions in panel (A). Each data point in panels A and B represents a unique donor organoid line (Donor ID 1, 2, and 5). (C) Relative TLR5 transcript levels detected in Donor ID 1 organoids stably transduced with three different shRNAs targeting TLR5 or a scrambled shRNA control. Organoids transduced with shRNA 2 were used in subsequent experiments. (D) Measurement of CXCL6 secreted to the basolateral medium of transduced organoid monolayers on permeable supports following 6 h infection with the indicated bacterial strains at 1 × 10^7^ CFU/mL inoculum. (E) Quantitative RT-PCR validation of epithelial transcripts detected by RNA-seq to demonstrate their dependence on expression of flagella/FliC in H10407 for induction. Each data point in panels D and E represents technical replicates using Donor ID 1 organoids; *n* = 3 independent experiments. Each graph depicts mean ± SEM. Statistical significance in each panel was calculated by one-way ANOVA with Dunnett’s correction for multiple comparisons, except for panel D, which was computed by two-way ANOVA with Tukey’s correction for multiple comparisons.

**FIG 3:**
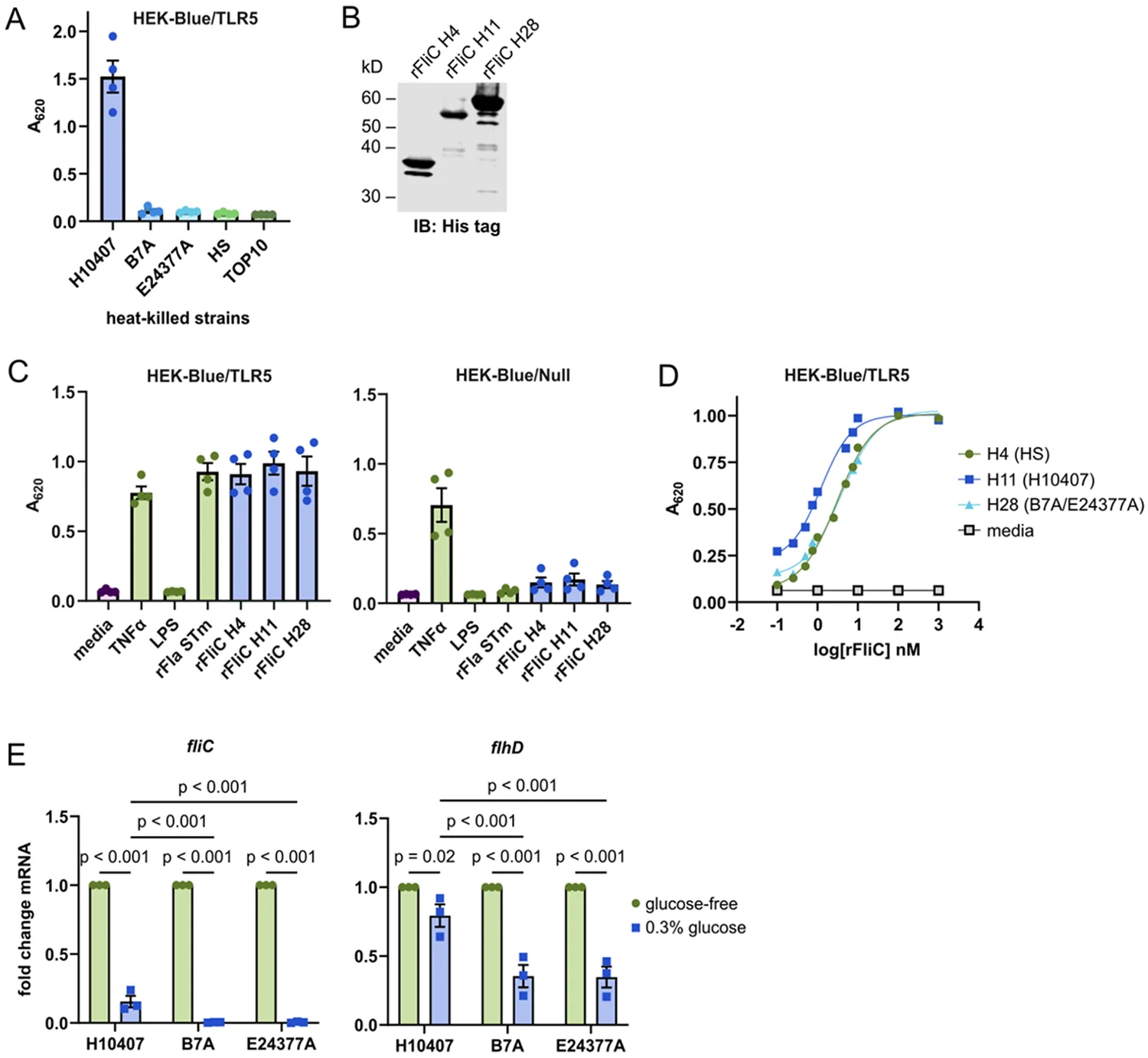
The flagellin monomer FliC in H10407 does not have differential TLR5 activation properties, but the flagellin operon appears to be differentially regulated. (A) Quanti-Blue absorbance measurements following overnight application of heat-killed bacterial strains to the engineered HEK293 reporter cell line HEK-Blue/hTLR5, which overexpresses human TLR5. *n* = 4 independent experiments. (B) Immunodetection of purified N-terminal 6×-histidine-tagged recombinant FliC (rFliC) proteins cloned from nonpathogenic *E. coli* HS (H4), H10407 (H11), or B7A/E24377A (H28). (C) HEK-Blue/hTLR5 and HEK-Blue/Null (lacks TLR5) reporter activation by recombinant FliC proteins (50 μM). rFla STm, recombinant flagellin from *Salmonella typhimurium*, a validated TLR5 ligand for the assay. TNFα was included as a TLR-independent ligand to demonstrate reporter activation. (D) Dose-response curves to calculate half-maximal effective concentrations (EC_50_) for HEK-Blue/hTLR5 reporter activation. Calculated EC_50_: rFliC H4 = 3.0 nM; rFliC H11 = 1.2 nM; rFliC H28 = 3.9 nM. (E) qRT-PCR of bacterial genes for *fliC* and the flagellar regulator *flhD* in strains grown in the absence or presence of glucose as an energy source. Each graph depicts mean ± SEM. Statistical significance was calculated by two-way ANOVA with Tukey’s correction for multiple comparisons.

**FIG 4:**
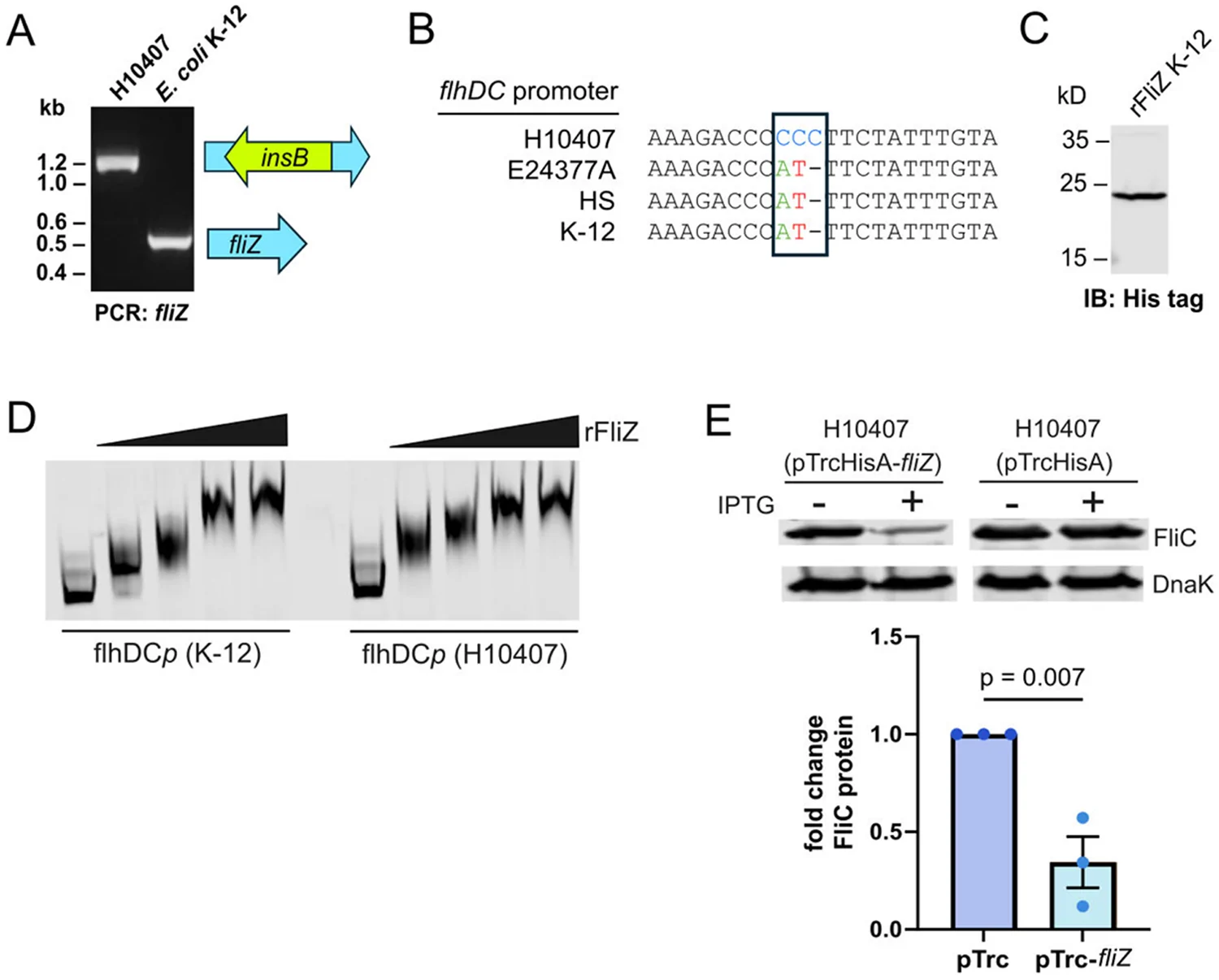
Loss of FliZ, and not mutations in the *flhDC* promoter, permits sustained flagellar expression in H10407. (A) PCR amplification of the *fliZ* gene using genomic DNA isolated from the respective strains. The *insB* insertion in H10407 introduces a frameshift and premature stop codon that fails to yield a functional protein. (B) Sequence alignment depicting *flhDC* promoter mutations in H10407 near the predicted binding site for FliZ. (C) Immunodetection of purified N-terminal 6×-histidine-tagged recombinant FliZ (rFliZ) protein produced using the gene sequence from *E. coli* K-12. (D) Representative EMSA using *flhDC* promoter fragments from the respective strains incubated in the presence of increasing amounts of rFliZ protein. (E) Representative immunoblot for FliC in H10407 carrying the *fliZ* complementation plasmid (pTrcHisA-fliZ) under control of a *lacI* promoter (IPTG-inducible). Empty plasmid (pTrcHisA) depicted as a negative control. DnaK was included as an endogenous loading control. Fold change in FliC protein under IPTG induction of FliZ expression was normalized to DnaK. *n* = 3 independent experiments. Graph depicts mean ± SEM. Statistical significance was calculated by Student’s *t* test.

**FIG 5:**
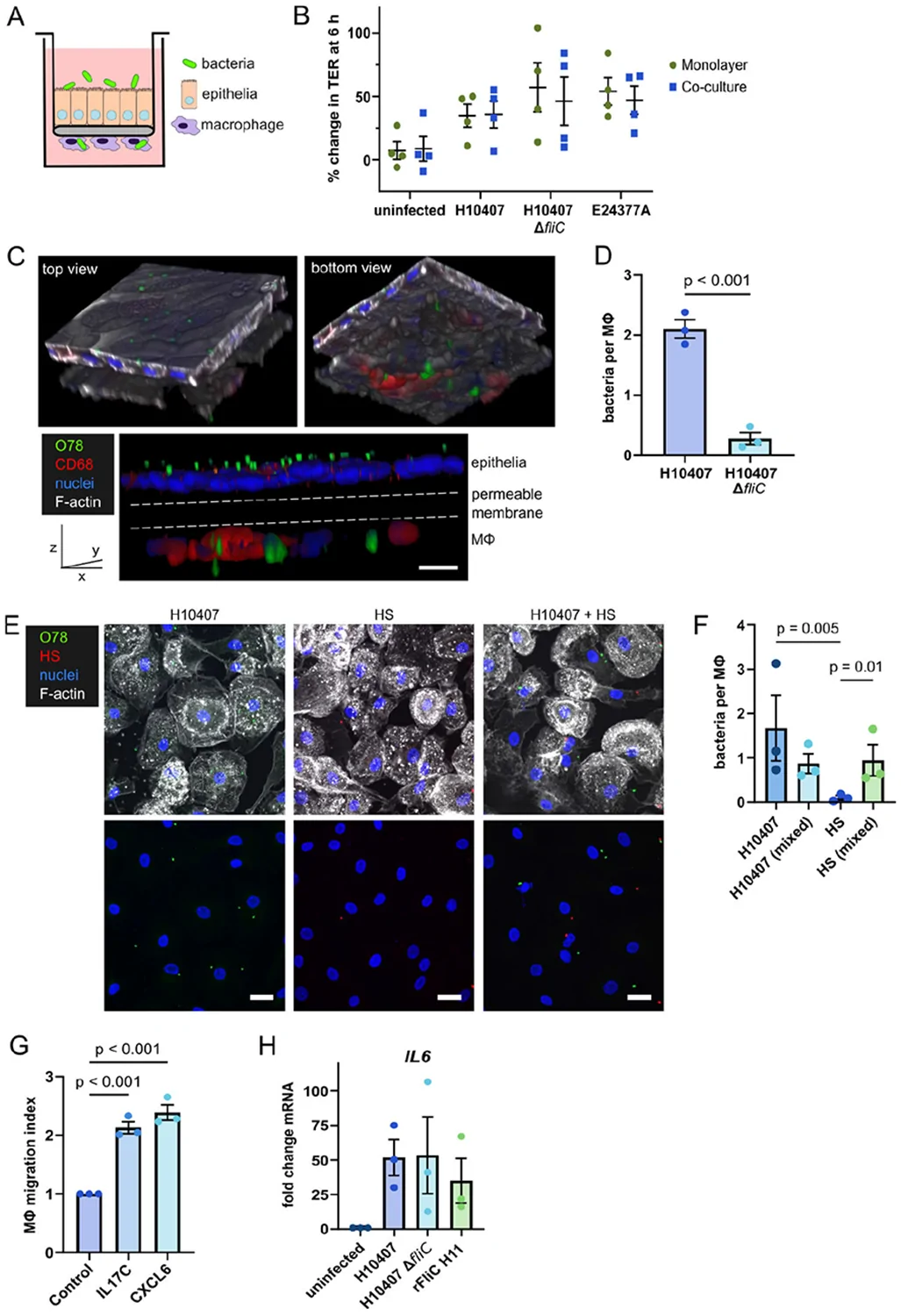
Flagellar expression promotes sampling of H10407 by macrophages co-cultured with epithelia. (A) Schematic of infected organoid monolayer-macrophage co-culture on a permeable support. (B) Percent change in TER after 6 h of infection in monolayer-only or co-culture experiments to demonstrate that the epithelial barrier remains intact. Each data point represents a unique donor organoid line (Donor IDs 1, 2, 5, and 6). For co-cultures, each organoid line was paired with a unique PBMC donor (IDs 3906, 5725, 6705, and 7396). (C) Z-projection confocal micrographs of monolayer-macrophage co-cultures infected with H10407 prior to fixation and immunostaining to show detection of bacteria (LPS antigen O78, green) at the apical epithelial surface (actin, white) as well as in association with/phagocytosed by macrophages (CD68, red; actin, white) on the opposite side of the 3.0-μm-pore membrane insert. In the bottom image, the F-actin channel has been computationally subtracted for clarity. Scale bar, 20 μm. (D) Ratio of bacteria per macrophage nucleus in infected co-cultures, enumerated by imaging and averaging eight random micrograph fields per condition, per independent experiment. *n* = 3 independent experiments using Donor ID 1 organoids paired with one of three PBMC donors (ID 5725, 6705, and 7396). (E) Representative images of the macrophage layer of co-cultures infected with 1 × 10^7^ CFU/mL of a single strain (H10407 or HS), or co-infected with equal numbers of H10407 and HS (each at 5 × 10^6^ CFU/mL) for 6 h. H10407 was detected using anti-O78, and HS was distinguished by expression of a plasmid-borne mScarlet-I reporter. Scale bar, 20 μm. (F) Ratio of the indicated bacterial strain counts per macrophage nucleus in co-cultures, analyzed as described for panel D. Each data point represents a unique organoid and PBMC donor pairing (IDs 1 with 7396, 3 with 6705, 6 with 5725). (G) Macrophage migration index for movement across an 8-μm-pore membrane seeded with macrophages on top and chemoattractant or vehicle (media) in the bottom chamber. *n* = 3 biological replicates (Donor IDs 3906, 6705, and 9215). (H) qRT-PCR comparison of *IL-6* transcripts in macrophages directly exposed to vehicle (media), rFliC protein (1 μg/mL), or bacteria (1 × 10^7^ CFU/mL) for 1 h. *n* = 3 biological replicates (Donor ID 3906, 6705, and 9215). Each graph depicts mean ± SEM. Statistics were calculated by two-tailed Student’s *t* test (D) or one-way ANOVA with Dunnett’s correction (F and G).

**FIG 6:**
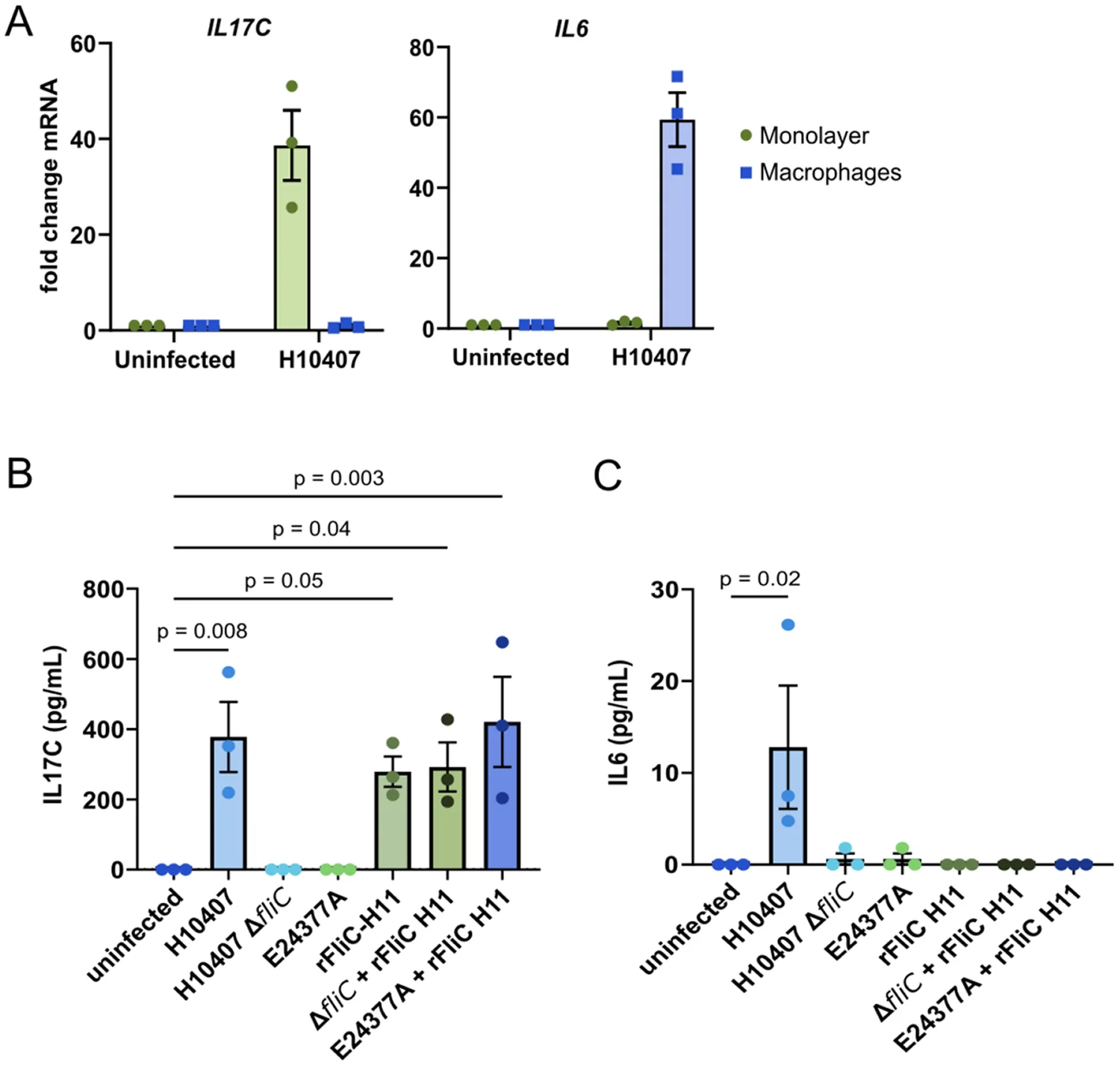
rFliC is sufficient to trigger cytokine release from epithelia, but endogenous FliC expression is required to stimulate macrophages. (A) qRT-PCR of directly infected monolayers (6 h) or macrophages (4 h) demonstrates that IL-17C originates from epithelia and IL-6 is induced in macrophages. Each data point is a technical replicate using 1 × 10^7^ CFU/mL bacteria and Donor ID 6 organoids or PBMC donor 7396, conducted in three independent experiments. (B) Measurement of IL-17C secreted by epithelia into the basolateral media of co-cultures infected with the respective strains and/or exposed to rFliC. (C) Measurement of IL-6 secreted by macrophages into the basolateral media under conditions equivalent to panel B. Each data point in panels B and C represents a co-culture of Donor ID 6 organoids paired with one of three PBMC donors (IDs 6705, 7396, and 9215). Each graph depicts mean ± SEM. Statistical significance was calculated by one-way ANOVA with Dunnett’s correction for multiple comparisons.

## Data Availability

RNA sequencing data generated and analyzed in this study are available for download from the Gene Expression Omnibus (GEO accession number GSE331118).
