# Supplementary material for: Flagellar regulation in enterotoxigenic E. coli modulates innate immune activation
Source: Infect Immun. Author manuscript; Available in PMC 2026 Oct 9. (PMC13647256; doi:10.1128/iai.00336-26)
Supplement: Supplementary file 1 [file NIHMS2211913-supplement-Supplementary_Materials.docx]

**SUPPLEMENTARY MATERIALS**

**Supplementary Table 1: Significant DEGs (|log2FC|≥1, *p*≤0.05), H10407 infection relative to uninfected control**

| **Gene symbol** | **Gene name** | **log2FC** | **-log10(pval)** |
| --- | --- | --- | --- |
| OLR1 | oxidized low density lipoprotein receptor 1 | 7.435 | 3.995 |
| TNFAIP6 | TNF alpha induced protein 6 | 7.430 | 3.765 |
| KIT | KIT proto-oncogene, receptor tyrosine kinase | 7.114 | 4.889 |
| CCL8 | C-C motif chemokine ligand 8 | 6.600 | 3.697 |
| IL17C | interleukin 17C | 6.542 | 11.863 |
| NOD2 | nucleotide binding oligomerization domain containing 2 | 5.468 | 5.818 |
| FEZ1 | fasciculation and elongation protein zeta 1 | 5.160 | 3.451 |
| C2CD4A | C2 calcium dependent domain containing 4A | 5.068 | 3.279 |
| PGLYRP2 | peptidoglycan recognition protein 2 | 4.890 | 4.367 |
| OVOS2 | alpha-2-macroglobulin like 1 pseudogene | 4.869 | 4.245 |
| HILPDA | hypoxia inducible lipid droplet associated | 4.772 | 6.472 |
| PTP4A3 | protein tyrosine phosphatase 4A3 | 4.744 | 2.327 |
| BCL2A1 | BCL2 related protein A1 | 4.438 | 6.141 |
| IL20 | interleukin 20 | 4.404 | 2.972 |
| IL19 | interleukin 19 | 4.363 | 1.769 |
| GFPT2 | glutamine-fructose-6-phosphate transaminase 2 | 4.206 | 4.409 |
| SNPH | syntaphilin | 4.044 | 6.017 |
| IL1A | interleukin 1 alpha | 3.928 | 4.455 |
| NOS2 | nitric oxide synthase 2 | 3.896 | 2.390 |
| WFDC5 | WAP four-disulfide core domain 5 | 3.849 | 3.308 |
| CD83 | CD83 molecule | 3.754 | 6.356 |
| PLA2G4E | phospholipase A2 group IVE | 3.697 | 2.477 |
| KRT6B | keratin 6B | 3.612 | 4.476 |
| SLFN11 | schlafen family member 11 | 3.497 | 1.621 |
| NKX3-1 | NK3 homeobox 1 | 3.433 | 4.092 |
| CXCL6 | C-X-C motif chemokine ligand 6 | 3.430 | 7.545 |
| NPHS1 | NPHS1 adhesion molecule, nephrin | 3.314 | 2.615 |
| TUBB2B | tubulin beta 2B class IIb | 3.310 | 2.784 |
| SLC43A3 | solute carrier family 43 member 3 | 3.290 | 2.745 |
| CCL5 | C-C motif chemokine ligand 5 | 3.247 | 1.940 |
| IL1B | interleukin 1 beta | 3.234 | 4.450 |
| SLC6A12 | solute carrier family 6 member 12 | 3.221 | 5.893 |
| CCL20 | C-C motif chemokine ligand 20 | 3.218 | 5.386 |
| G0S2 | G0/G1 switch 2 | 3.217 | 8.190 |
| TIFA | TRAF interacting protein with forkhead associated domain | 3.180 | 4.305 |
| SLC5A5 | solute carrier family 5 member 5 | 3.170 | 2.993 |
| CCL7 | C-C motif chemokine ligand 7 | 3.121 | 2.636 |
| SELE | selectin E | 3.068 | 1.636 |
| ZNF385C | zinc finger protein 385C | 3.052 | 6.300 |
| FMNL3 | formin like 3 | 3.031 | 6.613 |
| ARHGAP31 | Rho GTPase activating protein 31 | 3.029 | 5.054 |
| C2CD4B | C2 calcium dependent domain containing 4B | 2.960 | 2.190 |
| CLEC2D | C-type lectin domain family 2 member D | 2.909 | 4.164 |
| DOC2B | double C2 domain beta | 2.905 | 1.449 |
| REM2 | RRAD and GEM like GTPase 2 | 2.863 | 2.281 |
| CX3CL1 | C-X3-C motif chemokine ligand 1 | 2.859 | 3.830 |
| ICAM1 | intercellular adhesion molecule 1 | 2.856 | 7.268 |
| SOD2 | superoxide dismutase 2 | 2.844 | 7.449 |
| CXCL2 | C-X-C motif chemokine ligand 2 | 2.811 | 3.137 |
| TMOD2 | tropomodulin 2 | 2.800 | 2.521 |
| NEURL3 | neuralized E3 ubiquitin protein ligase 3 | 2.789 | 3.777 |
| UBASH3A | ubiquitin associated and SH3 domain containing A | 2.782 | 3.717 |
| CCL22 | C-C motif chemokine ligand 22 | 2.753 | 1.960 |
| RHCG | Rh family C glycoprotein | 2.738 | 4.321 |
| EBI3 | Epstein-Barr virus induced 3 | 2.728 | 2.213 |
| CXCL1 | C-X-C motif chemokine ligand 1 | 2.698 | 3.358 |
| ZC3H12A | zinc finger CCCH-type containing 12A | 2.691 | 7.530 |
| UNC13A | unc-13 homolog A | 2.644 | 4.442 |
| KIRREL2 | kirre like nephrin family adhesion molecule 2 | 2.643 | 1.476 |
| CSF2 | colony stimulating factor 2 | 2.619 | 4.263 |
| NPR1 | natriuretic peptide receptor 1 | 2.589 | 5.812 |
| NCCRP1 | NCCRP1, F-box associated domain containing | 2.568 | 2.103 |
| TNFRSF9 | TNF receptor superfamily member 9 | 2.532 | 4.730 |
| PTGS2 | prostaglandin-endoperoxide synthase 2 | 2.519 | 4.055 |
| RHOV | ras homolog family member V | 2.515 | 3.469 |
| LOC102723996 | ICOS ligand | 2.512 | 1.764 |
| CBFA2T3 | CBFA2/RUNX1 partner transcriptional co-repressor 3 | 2.388 | 2.767 |
| ICAM5 | intercellular adhesion molecule 5 | 2.386 | 2.641 |
| CARD14 | caspase recruitment domain family member 14 | 2.365 | 2.152 |
| SLC6A14 | solute carrier family 6 member 14 | 2.355 | 2.594 |
| FERMT3 | FERM domain containing kindlin 3 | 2.324 | 1.757 |
| BBC3 | BCL2 binding component 3 | 2.318 | 5.041 |
| ANKRD37 | ankyrin repeat domain 37 | 2.316 | 12.857 |
| SEMA3B-AS1 | SEMA3B antisense RNA 1 (head to head) | 2.312 | 1.604 |
| ITPRID1 | ITPR interacting domain containing 1 | 2.300 | 1.506 |
| NFKBIA | NFKB inhibitor alpha | 2.272 | 6.480 |
| RND1 | Rho family GTPase 1 | 2.267 | 4.815 |
| CHST2 | carbohydrate sulfotransferase 2 | 2.258 | 4.735 |
| IL18R1 | interleukin 18 receptor 1 | 2.232 | 2.559 |
| IL10RA | interleukin 10 receptor subunit alpha | 2.203 | 2.043 |
| NR4A1 | nuclear receptor subfamily 4 group A member 1 | 2.193 | 3.517 |
| SLC2A3 | solute carrier family 2 member 3 | 2.187 | 1.681 |
| CPNE5 | copine 5 | 2.174 | 2.705 |
| BPGM | bisphosphoglycerate mutase | 2.172 | 8.695 |
| DLL4 | delta like canonical Notch ligand 4 | 2.169 | 3.658 |
| SDC4 | syndecan 4 | 2.162 | 6.074 |
| CRABP2 | cellular retinoic acid binding protein 2 | 2.146 | 8.738 |
| CXCR4 | C-X-C motif chemokine receptor 4 | 2.140 | 11.812 |
| JCAD | junctional cadherin 5 associated | 2.124 | 2.366 |
| CXCL5 | C-X-C motif chemokine ligand 5 | 2.122 | 1.362 |
| SLCO4A1 | solute carrier organic anion transporter family member 4A1 | 2.099 | 5.470 |
| ADGRB2 | adhesion G protein-coupled receptor B2 | 2.092 | 1.491 |
| CXCL10 | C-X-C motif chemokine ligand 10 | 2.084 | 2.096 |
| GNA15 | G protein subunit alpha 15 | 2.069 | 1.985 |
| HMGB2 | high mobility group box 2 | 2.064 | 4.337 |
| CD69 | CD69 molecule | 2.053 | 1.491 |
| LOC105371730 | uncharacterized LOC105371730 | 2.042 | 1.888 |
| LINC00957 | long intergenic non-protein coding RNA 957 | 2.031 | 2.459 |
| ENKUR | enkurin, TRPC channel interacting protein | 2.021 | 1.575 |
| RASGEF1A | RasGEF domain family member 1A | 2.018 | 3.164 |
| DDIT4 | DNA damage inducible transcript 4 | 2.016 | 5.662 |
| TNFAIP3 | TNF alpha induced protein 3 | 1.999 | 5.914 |
| CXCL3 | C-X-C motif chemokine ligand 3 | 1.991 | 2.480 |
| BEAN1 | brain expressed associated with NEDD4 1 | 1.985 | 2.044 |
| BNIP5 | BCL2 interacting protein 5 | 1.984 | 4.197 |
| SH2D1B | SH2 domain containing 1B | 1.982 | 2.781 |
| MAP3K8 | mitogen-activated protein kinase kinase kinase 8 | 1.982 | 6.967 |
| TRAF1 | TNF receptor associated factor 1 | 1.982 | 5.907 |
| PRKD3 | protein kinase D3 | 1.980 | 4.042 |
| PIM3 | Pim-3 proto-oncogene, serine/threonine kinase | 1.961 | 7.863 |
| CEP112 | centrosomal protein 112 | 1.959 | 1.826 |
| RELT | RELT TNF receptor | 1.948 | 5.854 |
| C3 | complement C3 | 1.945 | 4.595 |
| DENND2B | DENN domain containing 2B | 1.940 | 4.457 |
| FOS | Fos proto-oncogene, AP-1 transcription factor subunit | 1.933 | 3.876 |
| NFATC1 | nuclear factor of activated T cells 1 | 1.921 | 1.999 |
| ICOSLG | inducible T cell costimulator ligand | 1.918 | 2.086 |
| CXCL8 | C-X-C motif chemokine ligand 8 | 1.917 | 5.110 |
| XDH | xanthine dehydrogenase | 1.916 | 4.735 |
| NAB2 | NGFI-A binding protein 2 | 1.916 | 3.907 |
| PRDM1 | PR/SET domain 1 | 1.899 | 2.380 |
| CDKN2C | cyclin dependent kinase inhibitor 2C | 1.899 | 2.760 |
| DNAH17 | dynein axonemal heavy chain 17 | 1.884 | 1.423 |
| C10orf55 | chromosome 10 putative open reading frame 55 | 1.878 | 1.926 |
| GDF15 | growth differentiation factor 15 | 1.876 | 2.181 |
| PLAT | plasminogen activator, tissue type | 1.852 | 2.049 |
| PSTPIP2 | proline-serine-threonine phosphatase interacting protein 2 | 1.829 | 3.463 |
| PTCH1 | patched 1 | 1.824 | 3.704 |
| TNFSF9 | TNF superfamily member 9 | 1.801 | 1.535 |
| PI3 | peptidase inhibitor 3 | 1.792 | 2.105 |
| RSRP1 | arginine and serine rich protein 1 | 1.788 | 4.983 |
| SERPINB7 | serpin family B member 7 | 1.785 | 1.495 |
| BIRC3 | baculoviral IAP repeat containing 3 | 1.784 | 5.569 |
| VEGFA | vascular endothelial growth factor A | 1.768 | 5.587 |
| RASGRP1 | RAS guanyl releasing protein 1 | 1.760 | 1.721 |
| IL23A | interleukin 23 subunit alpha | 1.758 | 4.717 |
| NAMPT | nicotinamide phosphoribosyltransferase | 1.755 | 3.270 |
| STAT5A | signal transducer and activator of transcription 5A | 1.747 | 3.753 |
| TNFAIP2 | TNF alpha induced protein 2 | 1.746 | 6.146 |
| WNT4 | Wnt family member 4 | 1.746 | 3.674 |
| BDKRB1 | bradykinin receptor B1 | 1.725 | 1.692 |
| EFNA3 | ephrin A3 | 1.721 | 3.924 |
| FOXA3 | forkhead box A3 | 1.715 | 5.281 |
| SOX7 | SRY-box transcription factor 7 | 1.704 | 3.157 |
| CD44 | CD44 molecule (Indian blood group) | 1.697 | 2.698 |
| N4BP3 | NEDD4 binding protein 3 | 1.696 | 2.417 |
| CSF1 | colony stimulating factor 1 | 1.676 | 3.155 |
| CHAC1 | ChaC glutathione specific gamma-glutamylcyclotransferase 1 | 1.661 | 3.931 |
| MMP25-AS1 | MMP25 antisense RNA 1 | 1.643 | 1.771 |
| ADM | adrenomedullin | 1.642 | 3.433 |
| GEM | GTP binding protein overexpressed in skeletal muscle | 1.639 | 4.071 |
| NFKB2 | nuclear factor kappa B subunit 2 | 1.638 | 6.848 |
| HLA-L | major histocompatibility complex, class I, L (pseudogene) | 1.638 | 1.594 |
| NIBAN1 | niban apoptosis regulator 1 | 1.631 | 1.579 |
| CDKN2D | cyclin dependent kinase inhibitor 2D | 1.630 | 5.804 |
| CA5B | carbonic anhydrase 5B | 1.621 | 2.619 |
| ENO2 | enolase 2 | 1.604 | 3.143 |
| PLEKHF1 | pleckstrin homology and FYVE domain containing 1 | 1.599 | 1.372 |
| CCSAP | centriole, cilia and spindle associated protein | 1.592 | 2.077 |
| SAT1 | spermidine/spermine N1-acetyltransferase 1 | 1.586 | 5.222 |
| WFDC3 | WAP four-disulfide core domain 3 | 1.541 | 1.701 |
| LOC101928596 | uncharacterized LOC101928596 | 1.539 | 2.959 |
| STX11 | syntaxin 11 | 1.524 | 2.437 |
| CHRNA5 | cholinergic receptor nicotinic alpha 5 subunit | 1.522 | 1.931 |
| GAS7 | growth arrest specific 7 | 1.506 | 4.109 |
| STARD5 | StAR related lipid transfer domain containing 5 | 1.501 | 3.948 |
| NOXO1 | NADPH oxidase organizer 1 | 1.500 | 2.646 |
| RORA | RAR related orphan receptor A | 1.499 | 2.069 |
| BAZ1A | bromodomain adjacent to zinc finger domain 1A | 1.498 | 2.849 |
| NFKBIZ | NFKB inhibitor zeta | 1.496 | 4.164 |
| RAB24 | RAB24, member RAS oncogene family | 1.495 | 4.154 |
| BCOR | BCL6 corepressor | 1.481 | 4.812 |
| EDN2 | endothelin 2 | 1.478 | 1.414 |
| DUSP10 | dual specificity phosphatase 10 | 1.475 | 2.845 |
| DUSP5 | dual specificity phosphatase 5 | 1.473 | 2.853 |
| CISH | cytokine inducible SH2 containing protein | 1.467 | 1.317 |
| SLC25A22 | solute carrier family 25 member 22 | 1.459 | 3.756 |
| MAFF | MAF bZIP transcription factor F | 1.454 | 3.971 |
| PFKFB3 | 6-phosphofructo-2-kinase/fructose-2,6-biphosphatase 3 | 1.449 | 3.286 |
| COL16A1 | collagen type XVI alpha 1 chain | 1.446 | 1.916 |
| B3GNT2 | UDP-GlcNAc:betaGal beta-1,3-N-acetylglucosaminyltransferase 2 | 1.444 | 1.758 |
| SIK1 | salt inducible kinase 1 | 1.439 | 5.745 |
| PIM1 | Pim-1 proto-oncogene, serine/threonine kinase | 1.439 | 4.600 |
| ANKRD36C | ankyrin repeat domain 36C | 1.438 | 1.523 |
| EFNA1 | ephrin A1 | 1.429 | 1.947 |
| APOBEC3G | apolipoprotein B mRNA editing enzyme catalytic subunit 3G | 1.428 | 1.570 |
| GCH1 | GTP cyclohydrolase 1 | 1.400 | 4.500 |
| WFDC12 | WAP four-disulfide core domain 12 | 1.396 | 1.374 |
| CCNE2 | cyclin E2 | 1.396 | 1.741 |
| PLAU | plasminogen activator, urokinase | 1.395 | 2.382 |
| HIF1A | hypoxia inducible factor 1 subunit alpha | 1.392 | 2.729 |
| PRSS53 | serine protease 53 | 1.387 | 1.301 |
| TNNT2 | troponin T2, cardiac type | 1.387 | 1.875 |
| PLEKHA4 | pleckstrin homology domain containing A4 | 1.385 | 1.528 |
| IFT57 | intraflagellar transport 57 | 1.378 | 3.363 |
| FRS3 | fibroblast growth factor receptor substrate 3 | 1.378 | 3.493 |
| NR4A2 | nuclear receptor subfamily 4 group A member 2 | 1.376 | 2.039 |
| ISG15 | ISG15 ubiquitin like modifier | 1.373 | 5.083 |
| STEAP4 | STEAP4 metalloreductase | 1.368 | 1.868 |
| CNOT8 | CCR4-NOT transcription complex subunit 8 | 1.363 | 2.781 |
| SLC28A3 | solute carrier family 28 member 3 | 1.362 | 2.460 |
| SLC25A48 | solute carrier family 25 member 48 | 1.360 | 1.387 |
| OGFRL1 | opioid growth factor receptor like 1 | 1.360 | 1.323 |
| COL9A2 | collagen type IX alpha 2 chain | 1.352 | 3.815 |
| FAM241A | family with sequence similarity 241 member A | 1.345 | 2.939 |
| ADAMTS9 | ADAM metallopeptidase with thrombospondin type 1 motif 9 | 1.344 | 3.225 |
| KHDRBS3 | KH RNA binding domain containing, signal transduction associated 3 | 1.339 | 1.336 |
| RAC2 | Rac family small GTPase 2 | 1.329 | 2.486 |
| FBXO32 | F-box protein 32 | 1.326 | 2.971 |
| RAB3A | RAB3A, member RAS oncogene family | 1.324 | 1.936 |
| TRIP10 | thyroid hormone receptor interactor 10 | 1.323 | 8.876 |
| DRAM1 | DNA damage regulated autophagy modulator 1 | 1.321 | 2.486 |
| LYSMD2 | LysM domain containing 2 | 1.313 | 1.436 |
| N4BP2L1 | NEDD4 binding protein 2 like 1 | 1.310 | 1.626 |
| RWDD2A | RWD domain containing 2A | 1.301 | 1.383 |
| ALCAM | activated leukocyte cell adhesion molecule | 1.282 | 1.857 |
| CYLD | CYLD lysine 63 deubiquitinase | 1.275 | 2.342 |
| NECTIN4 | nectin cell adhesion molecule 4 | 1.273 | 2.550 |
| EIF4A3 | eukaryotic translation initiation factor 4A3 | 1.270 | 3.050 |
| EFNA4 | ephrin A4 | 1.263 | 2.134 |
| ETV7 | ETS variant transcription factor 7 | 1.263 | 2.050 |
| ERO1A | endoplasmic reticulum oxidoreductase 1 alpha | 1.262 | 1.932 |
| VPS37D | VPS37D subunit of ESCRT-I | 1.261 | 1.690 |
| NFKBIE | NFKB inhibitor epsilon | 1.258 | 2.723 |
| ARRDC1-AS1 | ARRDC1 antisense RNA 1 | 1.253 | 2.036 |
| TBC1D10A | TBC1 domain family member 10A | 1.250 | 6.939 |
| CLK3 | CDC like kinase 3 | 1.245 | 6.226 |
| BCL2L11 | BCL2 like 11 | 1.243 | 2.549 |
| PHF20 | PHD finger protein 20 | 1.235 | 4.542 |
| SERTAD2 | SERTA domain containing 2 | 1.231 | 1.943 |
| TBC1D2B | TBC1 domain family member 2B | 1.231 | 2.375 |
| HIVEP2 | HIVEP zinc finger 2 | 1.228 | 2.480 |
| ACYP1 | acylphosphatase 1 | 1.224 | 1.345 |
| PIGA | phosphatidylinositol glycan anchor biosynthesis class A | 1.223 | 2.707 |
| RAPGEF3 | Rap guanine nucleotide exchange factor 3 | 1.221 | 1.788 |
| BATF2 | basic leucine zipper ATF-like transcription factor 2 | 1.212 | 2.206 |
| CLK1 | CDC like kinase 1 | 1.211 | 4.724 |
| JAK2 | Janus kinase 2 | 1.210 | 2.423 |
| RHBDF2 | rhomboid 5 homolog 2 | 1.209 | 1.978 |
| PIM2 | Pim-2 proto-oncogene, serine/threonine kinase | 1.187 | 1.347 |
| RNF223 | ring finger protein 223 | 1.183 | 1.325 |
| TNFRSF1B | TNF receptor superfamily member 1B | 1.174 | 2.562 |
| LYN | LYN proto-oncogene, Src family tyrosine kinase | 1.170 | 2.481 |
| RALGDS | ral guanine nucleotide dissociation stimulator | 1.169 | 2.576 |
| BTG3 | BTG anti-proliferation factor 3 | 1.169 | 1.914 |
| OAF | out at first homolog | 1.169 | 3.893 |
| TNIP1 | TNFAIP3 interacting protein 1 | 1.168 | 3.338 |
| FAM210A | family with sequence similarity 210 member A | 1.167 | 4.361 |
| MXD1 | MAX dimerization protein 1 | 1.166 | 2.257 |
| PER1 | period circadian regulator 1 | 1.165 | 2.083 |
| AVPI1 | arginine vasopressin induced 1 | 1.163 | 5.889 |
| LOC102724428 | salt inducible kinase 1B (putative) | 1.160 | 1.715 |
| DCUN1D3 | defective in cullin neddylation 1 domain containing 3 | 1.160 | 3.500 |
| SAV1 | salvador family WW domain containing protein 1 | 1.159 | 1.776 |
| CDKN1A | cyclin dependent kinase inhibitor 1A | 1.156 | 1.857 |
| S100A3 | S100 calcium binding protein A3 | 1.154 | 1.547 |
| BID | BH3 interacting domain death agonist | 1.154 | 4.932 |
| PPP4R2 | protein phosphatase 4 regulatory subunit 2 | 1.150 | 3.823 |
| ANKRD36 | ankyrin repeat domain 36 | 1.149 | 2.480 |
| DNTTIP1 | deoxynucleotidyltransferase terminal interacting protein 1 | 1.148 | 2.737 |
| IL32 | interleukin 32 | 1.148 | 2.449 |
| POU2F2 | POU class 2 homeobox 2 | 1.142 | 1.386 |
| RSRC2 | arginine and serine rich coiled-coil 2 | 1.139 | 3.779 |
| ISG20 | interferon stimulated exonuclease gene 20 | 1.138 | 2.569 |
| SMNDC1 | survival motor neuron domain containing 1 | 1.135 | 3.384 |
| ANKRD36B | ankyrin repeat domain 36B | 1.133 | 2.330 |
| TMPRSS2 | transmembrane serine protease 2 | 1.130 | 1.542 |
| BCL6 | BCL6 transcription repressor | 1.125 | 1.779 |
| TRERF1 | transcriptional regulating factor 1 | 1.125 | 1.917 |
| BARX2 | BARX homeobox 2 | 1.124 | 1.350 |
| HK2 | hexokinase 2 | 1.124 | 1.307 |
| REL | REL proto-oncogene, NF-kB subunit | 1.118 | 2.285 |
| NFKB1 | nuclear factor kappa B subunit 1 | 1.114 | 2.622 |
| ZFP36 | ZFP36 ring finger protein | 1.112 | 1.433 |
| PSMA6 | proteasome 20S subunit alpha 6 | 1.111 | 2.623 |
| CENPW | centromere protein W | 1.109 | 1.348 |
| ANGPTL4 | angiopoietin like 4 | 1.107 | 2.460 |
| LNX1 | ligand of numb-protein X 1 | 1.105 | 2.473 |
| CSRNP1 | cysteine and serine rich nuclear protein 1 | 1.104 | 4.210 |
| ST3GAL1 | ST3 beta-galactoside alpha-2,3-sialyltransferase 1 | 1.104 | 2.829 |
| BHLHE40 | basic helix-loop-helix family member e40 | 1.103 | 2.961 |
| TMEM106A | transmembrane protein 106A | 1.101 | 1.533 |
| IKBKE | inhibitor of nuclear factor kappa B kinase subunit epsilon | 1.070 | 2.994 |
| TMEM74B | transmembrane protein 74B | 1.070 | 3.163 |
| RILPL2 | Rab interacting lysosomal protein like 2 | 1.067 | 1.415 |
| PTMS | parathymosin | 1.067 | 2.931 |
| SERTAD3 | SERTA domain containing 3 | 1.064 | 2.373 |
| TIPARP | TCDD inducible poly(ADP-ribose) polymerase | 1.061 | 2.309 |
| ZHX2 | zinc fingers and homeoboxes 2 | 1.061 | 2.347 |
| ING1 | inhibitor of growth family member 1 | 1.053 | 1.914 |
| ATP6V1C2 | ATPase H+ transporting V1 subunit C2 | 1.047 | 2.158 |
| BTN2A3P | butyrophilin subfamily 2 member A3, pseudogene | 1.045 | 2.370 |
| PLAUR | plasminogen activator, urokinase receptor | 1.041 | 1.955 |
| SLC2A6 | solute carrier family 2 member 6 | 1.040 | 1.372 |
| DPM3 | dolichyl-phosphate mannosyltransferase subunit 3, regulatory | 1.033 | 1.439 |
| FAM219A | family with sequence similarity 219 member A | 1.031 | 1.858 |
| NUB1 | negative regulator of ubiquitin like proteins 1 | 1.031 | 2.217 |
| SLC25A25 | solute carrier family 25 member 25 | 1.010 | 1.577 |
| GCKR | glucokinase regulator | 1.009 | 2.032 |
| TNFSF10 | TNF superfamily member 10 | 1.008 | 2.618 |
| ZNF622 | zinc finger protein 622 | 1.008 | 2.613 |
| RELB | RELB proto-oncogene, NF-kB subunit | 1.007 | 2.705 |
| PELI1 | pellino E3 ubiquitin protein ligase 1 | 1.006 | 2.587 |
| RAP1GAP | RAP1 GTPase activating protein | 1.006 | 1.809 |
| MMP10 | matrix metallopeptidase 10 | 1.003 | 1.369 |
| CCNG1 | cyclin G1 | 1.002 | 2.419 |
| MRTO4 | MRT4 homolog, ribosome maturation factor | -1.001 | 2.186 |
| KRT80 | keratin 80 | -1.002 | 2.562 |
| TCEANC | transcription elongation factor A N-terminal and central domain containing | -1.005 | 1.705 |
| ZNF678 | zinc finger protein 678 | -1.014 | 1.563 |
| PCNX3 | pecanex 3 | -1.015 | 2.031 |
| SFT2D2 | SFT2 domain containing 2 | -1.020 | 4.644 |
| METTL1 | methyltransferase 1, tRNA methylguanosine | -1.021 | 1.323 |
| MTCL2 | microtubule crosslinking factor 2 | -1.022 | 2.507 |
| CDC42EP3 | CDC42 effector protein 3 | -1.023 | 1.329 |
| HGH1 | HGH1 homolog | -1.025 | 1.592 |
| HSPA8 | heat shock protein family A (Hsp70) member 8 | -1.028 | 4.983 |
| PHETA2 | PH domain containing endocytic trafficking adaptor 2 | -1.033 | 1.863 |
| NBPF26 | NBPF member 26 | -1.033 | 2.147 |
| IQCE | IQ motif containing E | -1.036 | 2.679 |
| TNS3 | tensin 3 | -1.036 | 2.947 |
| CEP78 | centrosomal protein 78 | -1.036 | 3.357 |
| SLC35B4 | solute carrier family 35 member B4 | -1.042 | 3.000 |
| ZBED4 | zinc finger BED-type containing 4 | -1.042 | 1.495 |
| POLR1B | RNA polymerase I subunit B | -1.043 | 1.714 |
| CCNJ | cyclin J | -1.043 | 2.414 |
| USP46 | ubiquitin specific peptidase 46 | -1.043 | 2.592 |
| NACC2 | NACC family member 2 | -1.044 | 3.165 |
| TNRC6B | trinucleotide repeat containing adaptor 6B | -1.044 | 4.159 |
| FOXA1 | forkhead box A1 | -1.044 | 1.950 |
| NR2C1 | nuclear receptor subfamily 2 group C member 1 | -1.045 | 4.710 |
| LINC01269 | long intergenic non-protein coding RNA 1269 | -1.045 | 1.434 |
| GTF2E1 | general transcription factor IIE subunit 1 | -1.049 | 2.560 |
| LAMA5 | laminin subunit alpha 5 | -1.049 | 1.552 |
| BBS5 | Bardet-Biedl syndrome 5 | -1.052 | 1.362 |
| CASC19 | cancer susceptibility 19 | -1.058 | 1.647 |
| TP53INP1 | tumor protein p53 inducible nuclear protein 1 | -1.061 | 1.850 |
| CASTOR3P | CASTOR family member 3, pseudogene | -1.061 | 2.319 |
| KANSL3 | KAT8 regulatory NSL complex subunit 3 | -1.062 | 4.096 |
| ARHGAP29 | Rho GTPase activating protein 29 | -1.063 | 1.659 |
| FBXO41 | F-box protein 41 | -1.064 | 1.534 |
| TACC3 | transforming acidic coiled-coil containing protein 3 | -1.065 | 4.607 |
| SBF1 | SET binding factor 1 | -1.065 | 4.201 |
| GNAL | G protein subunit alpha L | -1.068 | 2.437 |
| FBXO9 | F-box protein 9 | -1.070 | 5.000 |
| NOD1 | nucleotide binding oligomerization domain containing 1 | -1.081 | 1.514 |
| ARL4A | ADP ribosylation factor like GTPase 4A | -1.082 | 1.801 |
| DEPDC7 | DEP domain containing 7 | -1.091 | 1.626 |
| BYSL | bystin like | -1.093 | 2.072 |
| DCAF4 | DDB1 and CUL4 associated factor 4 | -1.095 | 3.809 |
| B3GALT5 | beta-1,3-galactosyltransferase 5 | -1.098 | 2.524 |
| ZXDC | ZXD family zinc finger C | -1.099 | 3.715 |
| ZNF689 | zinc finger protein 689 | -1.100 | 2.364 |
| ZNF202 | zinc finger protein 202 | -1.100 | 1.311 |
| ZNF362 | zinc finger protein 362 | -1.102 | 1.877 |
| CCDC171 | coiled-coil domain containing 171 | -1.105 | 1.997 |
| EXTL3 | exostosin like glycosyltransferase 3 | -1.105 | 1.780 |
| SARM1 | sterile alpha and TIR motif containing 1 | -1.106 | 1.628 |
| PTPRU | protein tyrosine phosphatase receptor type U | -1.110 | 1.544 |
| CDCA7L | cell division cycle associated 7 like | -1.110 | 1.914 |
| NF2 | NF2, moesin-ezrin-radixin like (MERLIN) tumor suppressor | -1.110 | 1.932 |
| NBEAL2 | neurobeachin like 2 | -1.115 | 3.002 |
| PITPNM3 | PITPNM family member 3 | -1.124 | 2.594 |
| MICAL2 | microtubule associated monooxygenase, calponin and LIM domain containing 2 | -1.125 | 3.720 |
| NBPF1 | NBPF member 1 | -1.129 | 1.370 |
| ZNF605 | zinc finger protein 605 | -1.130 | 1.762 |
| CDON | cell adhesion associated, oncogene regulated | -1.136 | 1.584 |
| CCDC142 | coiled-coil domain containing 142 | -1.139 | 2.660 |
| MYOM3 | myomesin 3 | -1.140 | 2.087 |
| C21orf91 | chromosome 21 open reading frame 91 | -1.141 | 1.500 |
| ISX | intestine specific homeobox | -1.142 | 4.900 |
| DDX31 | DEAD-box helicase 31 | -1.144 | 1.595 |
| ZNF75A | zinc finger protein 75A | -1.144 | 1.894 |
| ZSCAN20 | zinc finger and SCAN domain containing 20 | -1.152 | 5.272 |
| NBPF12 | NBPF member 12 | -1.155 | 6.141 |
| CEP76 | centrosomal protein 76 | -1.156 | 2.467 |
| PKD1 | polycystin 1, transient receptor potential channel interacting | -1.163 | 2.493 |
| ANKS6 | ankyrin repeat and sterile alpha motif domain containing 6 | -1.166 | 1.707 |
| RBM12B | RNA binding motif protein 12B | -1.171 | 1.537 |
| TLN2 | talin 2 | -1.172 | 2.020 |
| TRIM16L | tripartite motif containing 16 like (pseudogene) | -1.176 | 4.116 |
| ZNF225 | zinc finger protein 225 | -1.178 | 1.831 |
| NT5DC2 | 5'-nucleotidase domain containing 2 | -1.182 | 1.712 |
| DHX35 | DEAH-box helicase 35 | -1.183 | 3.509 |
| FAM217B | family with sequence similarity 217 member B | -1.185 | 2.317 |
| ZNF180 | zinc finger protein 180 | -1.185 | 3.039 |
| SUV39H1 | SUV39H1 histone lysine methyltransferase | -1.187 | 1.939 |
| ZNF35 | zinc finger protein 35 | -1.190 | 1.702 |
| CLDN10-AS1 | CLDN10 antisense RNA 1 | -1.193 | 1.324 |
| PFAS | phosphoribosylformylglycinamidine synthase | -1.193 | 1.873 |
| POLR3H | RNA polymerase III subunit H | -1.200 | 2.045 |
| PLCXD2 | phosphatidylinositol specific phospholipase C X domain containing 2 | -1.203 | 2.447 |
| CDR2 | cerebellar degeneration related protein 2 | -1.204 | 3.694 |
| TRMT5 | tRNA methyltransferase 5 | -1.210 | 3.197 |
| MFNG | MFNG O-fucosylpeptide 3-beta-N-acetylglucosaminyltransferase | -1.213 | 1.655 |
| ZNF786 | zinc finger protein 786 | -1.213 | 1.843 |
| GEN1 | GEN1 Holliday junction 5' flap endonuclease | -1.219 | 3.804 |
| RAB3D | RAB3D, member RAS oncogene family | -1.219 | 1.514 |
| WARS2-AS1 | WARS2 antisense RNA 1 | -1.220 | 1.720 |
| KNOP1 | lysine rich nucleolar protein 1 | -1.220 | 2.017 |
| KITLG | KIT ligand | -1.220 | 1.455 |
| SRXN1 | sulfiredoxin 1 | -1.221 | 1.544 |
| PHETA1 | PH domain containing endocytic trafficking adaptor 1 | -1.223 | 1.531 |
| ZNF8 | zinc finger protein 8 | -1.224 | 2.134 |
| HIRA | histone cell cycle regulator | -1.225 | 2.914 |
| VANGL1 | VANGL planar cell polarity protein 1 | -1.225 | 3.650 |
| NCS1 | neuronal calcium sensor 1 | -1.236 | 1.555 |
| RBM4B | RNA binding motif protein 4B | -1.236 | 3.373 |
| NHSL1 | NHS like 1 | -1.237 | 4.041 |
| PLEKHA8P1 | pleckstrin homology domain containing A8 pseudogene 1 | -1.239 | 2.003 |
| POLR1A | RNA polymerase I subunit A | -1.245 | 2.233 |
| SLC29A3 | solute carrier family 29 member 3 | -1.247 | 2.196 |
| EPC1 | enhancer of polycomb homolog 1 | -1.248 | 8.218 |
| LMO4 | LIM domain only 4 | -1.254 | 2.028 |
| CELSR1 | cadherin EGF LAG seven-pass G-type receptor 1 | -1.260 | 2.489 |
| MTARC1 | mitochondrial amidoxime reducing component 1 | -1.261 | 1.780 |
| STON2 | stonin 2 | -1.264 | 1.520 |
| CBX6 | chromobox 6 | -1.267 | 4.222 |
| SRGAP1 | SLIT-ROBO Rho GTPase activating protein 1 | -1.276 | 5.662 |
| TSEN2 | tRNA splicing endonuclease subunit 2 | -1.282 | 3.202 |
| ZNF302 | zinc finger protein 302 | -1.285 | 1.832 |
| NIPAL1 | NIPA like domain containing 1 | -1.285 | 4.263 |
| BAMBI | BMP and activin membrane bound inhibitor | -1.287 | 2.415 |
| LTO1 | LTO1 maturation factor of ABCE1 | -1.288 | 4.652 |
| ADCY7 | adenylate cyclase 7 | -1.292 | 2.303 |
| MEPCE | methylphosphate capping enzyme | -1.294 | 2.248 |
| GCLC | glutamate-cysteine ligase catalytic subunit | -1.294 | 2.521 |
| BICD2 | BICD cargo adaptor 2 | -1.294 | 5.757 |
| LRRC20 | leucine rich repeat containing 20 | -1.294 | 4.006 |
| HDAC4 | histone deacetylase 4 | -1.298 | 4.680 |
| WDR4 | WD repeat domain 4 | -1.300 | 1.463 |
| SPRY2 | sprouty RTK signaling antagonist 2 | -1.302 | 2.630 |
| PLK2 | polo like kinase 2 | -1.309 | 6.848 |
| C11orf86 | chromosome 11 open reading frame 86 | -1.321 | 3.055 |
| MAVS | mitochondrial antiviral signaling protein | -1.321 | 3.889 |
| ATXN7L2 | ataxin 7 like 2 | -1.326 | 3.385 |
| MMP24 | matrix metallopeptidase 24 | -1.326 | 1.580 |
| RARB | retinoic acid receptor beta | -1.329 | 3.106 |
| COQ10A | coenzyme Q10A | -1.331 | 1.416 |
| EXO5 | exonuclease 5 | -1.332 | 1.750 |
| FAM168B | family with sequence similarity 168 member B | -1.334 | 1.640 |
| SPTBN2 | spectrin beta, non-erythrocytic 2 | -1.339 | 3.374 |
| CENPL | centromere protein L | -1.353 | 1.800 |
| SLC7A11 | solute carrier family 7 member 11 | -1.365 | 1.694 |
| MTERF1 | mitochondrial transcription termination factor 1 | -1.366 | 1.532 |
| ZBTB3 | zinc finger and BTB domain containing 3 | -1.368 | 1.402 |
| ACACB | acetyl-CoA carboxylase beta | -1.371 | 4.069 |
| XRCC3 | X-ray repair cross complementing 3 | -1.374 | 1.867 |
| L3HYPDH | trans-L-3-hydroxyproline dehydratase | -1.377 | 1.929 |
| SORCS2 | sortilin related VPS10 domain containing receptor 2 | -1.377 | 1.360 |
| TBC1D13 | TBC1 domain family member 13 | -1.378 | 3.308 |
| PCF11 | PCF11 cleavage and polyadenylation factor subunit | -1.384 | 5.857 |
| TNS4 | tensin 4 | -1.385 | 1.466 |
| PSKH1 | protein serine kinase H1 | -1.390 | 1.683 |
| CMTM4 | CKLF like MARVEL transmembrane domain containing 4 | -1.402 | 2.047 |
| XYLT1 | xylosyltransferase 1 | -1.406 | 3.811 |
| ZNF772 | zinc finger protein 772 | -1.412 | 1.537 |
| OSGIN1 | oxidative stress induced growth inhibitor 1 | -1.420 | 1.379 |
| LRRC66 | leucine rich repeat containing 66 | -1.420 | 3.193 |
| LCAT | lecithin-cholesterol acyltransferase | -1.421 | 1.697 |
| USP22 | ubiquitin specific peptidase 22 | -1.425 | 3.676 |
| DOK7 | docking protein 7 | -1.427 | 2.590 |
| DTX4 | deltex E3 ubiquitin ligase 4 | -1.428 | 3.145 |
| KRT10 | keratin 10 | -1.431 | 1.832 |
| CEL | carboxyl ester lipase | -1.435 | 1.432 |
| UGT1A6 | UDP glucuronosyltransferase family 1 member A6 | -1.447 | 1.526 |
| ZNF888 | zinc finger protein 888 | -1.448 | 1.910 |
| NDST1 | N-deacetylase and N-sulfotransferase 1 | -1.450 | 5.134 |
| TRAM2 | translocation associated membrane protein 2 | -1.451 | 2.023 |
| LINC00857 | long intergenic non-protein coding RNA 857 | -1.456 | 2.495 |
| HPX | hemopexin | -1.469 | 1.570 |
| MDN1 | midasin AAA ATPase 1 | -1.469 | 9.367 |
| MLXIP | MLX interacting protein | -1.471 | 3.277 |
| WDR37 | WD repeat domain 37 | -1.480 | 6.932 |
| URB2 | URB2 ribosome biogenesis homolog | -1.481 | 2.681 |
| MAT2A | methionine adenosyltransferase 2A | -1.485 | 4.286 |
| MEGF8 | multiple EGF like domains 8 | -1.488 | 3.333 |
| THBS1 | thrombospondin 1 | -1.488 | 2.480 |
| ANKRD52 | ankyrin repeat domain 52 | -1.497 | 3.342 |
| NWD1 | NACHT and WD repeat domain containing 1 | -1.499 | 2.533 |
| DANCR | differentiation antagonizing non-protein coding RNA | -1.499 | 1.459 |
| HR | HR lysine demethylase and nuclear receptor corepressor | -1.504 | 2.594 |
| CYTH3 | cytohesin 3 | -1.505 | 5.783 |
| ZC3H8 | zinc finger CCCH-type containing 8 | -1.509 | 2.974 |
| ASTE1 | asteroid homolog 1 | -1.511 | 5.064 |
| NCOA5 | nuclear receptor coactivator 5 | -1.531 | 8.870 |
| TRIM25 | tripartite motif containing 25 | -1.532 | 3.482 |
| PRDM11 | PR/SET domain 11 | -1.541 | 2.637 |
| TBC1D16 | TBC1 domain family member 16 | -1.543 | 6.108 |
| URB1 | URB1 ribosome biogenesis homolog | -1.550 | 3.074 |
| EFNB2 | ephrin B2 | -1.557 | 2.828 |
| ADSS1 | adenylosuccinate synthase 1 | -1.557 | 4.390 |
| KIF23 | kinesin family member 23 | -1.559 | 1.959 |
| C1orf226 | chromosome 1 open reading frame 226 | -1.562 | 3.927 |
| ARAP3 | ArfGAP with RhoGAP domain, ankyrin repeat and PH domain 3 | -1.573 | 4.381 |
| KDM8 | lysine demethylase 8 | -1.590 | 5.189 |
| CABLES1 | Cdk5 and Abl enzyme substrate 1 | -1.591 | 4.406 |
| CCDC113 | coiled-coil domain containing 113 | -1.594 | 1.609 |
| ING3 | inhibitor of growth family member 3 | -1.596 | 7.137 |
| DGKD | diacylglycerol kinase delta | -1.598 | 3.924 |
| BCDIN3D | BCDIN3 domain containing RNA methyltransferase | -1.599 | 1.665 |
| MCM3AP-AS1 | MCM3AP antisense RNA 1 | -1.599 | 1.538 |
| PPARGC1B | PPARG coactivator 1 beta | -1.630 | 1.326 |
| LMCD1 | LIM and cysteine rich domains 1 | -1.639 | 2.193 |
| TRIB1 | tribbles pseudokinase 1 | -1.641 | 3.527 |
| CYP26B1 | cytochrome P450 family 26 subfamily B member 1 | -1.653 | 1.405 |
| ADAMTS15 | ADAM metallopeptidase with thrombospondin type 1 motif 15 | -1.654 | 1.496 |
| TWNK | twinkle mtDNA helicase | -1.671 | 1.494 |
| TMEM131L | transmembrane 131 like | -1.686 | 2.943 |
| KAT14 | lysine acetyltransferase 14 | -1.686 | 7.076 |
| EPHA4 | EPH receptor A4 | -1.689 | 3.008 |
| TOE1 | target of EGR1, exonuclease | -1.703 | 6.239 |
| ADGRF5 | adhesion G protein-coupled receptor F5 | -1.711 | 2.081 |
| PCED1B | PC-esterase domain containing 1B | -1.742 | 1.358 |
| PIK3CD | phosphatidylinositol-4,5-bisphosphate 3-kinase catalytic subunit delta | -1.745 | 3.044 |
| PIH1D2 | PIH1 domain containing 2 | -1.766 | 1.437 |
| NUAK1 | NUAK family kinase 1 | -1.768 | 3.670 |
| SPATA33 | spermatogenesis associated 33 | -1.802 | 1.577 |
| ENC1 | ectodermal-neural cortex 1 | -1.857 | 2.282 |
| HMGA2 | high mobility group AT-hook 2 | -1.859 | 1.513 |
| ITIH4 | inter-alpha-trypsin inhibitor heavy chain 4 | -1.862 | 1.365 |
| COMETT | cytosolic oncogenic antisense to MET transcript | -1.862 | 1.676 |
| CENPO | centromere protein O | -1.871 | 5.592 |
| CELSR3 | cadherin EGF LAG seven-pass G-type receptor 3 | -1.899 | 2.154 |
| TXK | TXK tyrosine kinase | -1.915 | 2.091 |
| RGS2 | regulator of G protein signaling 2 | -1.917 | 1.610 |
| MITF | melanocyte inducing transcription factor | -1.922 | 1.956 |
| RGS5 | regulator of G protein signaling 5 | -1.926 | 2.361 |
| MGAT3 | beta-1,4-mannosyl-glycoprotein 4-beta-N-acetylglucosaminyltransferase | -1.931 | 2.458 |
| AVIL | advillin | -1.936 | 3.193 |
| HCN4 | hyperpolarization activated cyclic nucleotide gated potassium channel 4 | -1.945 | 1.634 |
| IFFO2 | intermediate filament family orphan 2 | -1.950 | 6.712 |
| ZSCAN31 | zinc finger and SCAN domain containing 31 | -1.951 | 3.841 |
| BAZ2B-AS1 | BAZ2B antisense RNA 1 | -2.004 | 1.464 |
| EFR3B | EFR3 homolog B | -2.006 | 2.781 |
| PSMG3-AS1 | PSMG3 antisense RNA 1 (head to head) | -2.009 | 3.222 |
| MEF2C | myocyte enhancer factor 2C | -2.017 | 4.701 |
| ARHGEF37 | Rho guanine nucleotide exchange factor 37 | -2.030 | 5.830 |
| FRMPD1 | FERM and PDZ domain containing 1 | -2.056 | 2.132 |
| FAM13B | family with sequence similarity 13 member B | -2.058 | 10.188 |
| SLC22A20P | solute carrier family 22 member 20, pseudogene | -2.066 | 1.881 |
| LINC01588 | long intergenic non-protein coding RNA 1588 | -2.074 | 2.947 |
| CCAT1 | colon cancer associated transcript 1 | -2.091 | 1.726 |
| FDXACB1 | ferredoxin-fold anticodon binding domain containing 1 | -2.129 | 2.539 |
| GRAMD1B | GRAM domain containing 1B | -2.135 | 6.399 |
| CAD | carbamoyl-phosphate synthetase 2, aspartate transcarbamylase, and dihydroorotase | -2.137 | 4.391 |
| DTNA | dystrobrevin alpha | -2.154 | 1.381 |
| ZNF696 | zinc finger protein 696 | -2.154 | 2.333 |
| UNC119B | unc-119 lipid binding chaperone B | -2.160 | 4.444 |
| PHACTR3 | phosphatase and actin regulator 3 | -2.160 | 2.144 |
| ANKRD44 | ankyrin repeat domain 44 | -2.225 | 1.870 |
| PDE7B | phosphodiesterase 7B | -2.230 | 2.271 |
| EGR1 | early growth response 1 | -2.246 | 1.327 |
| CYB561D1 | cytochrome b561 family member D1 | -2.267 | 4.502 |
| HS3ST5 | heparan sulfate-glucosamine 3-sulfotransferase 5 | -2.308 | 1.318 |
| FAM53B | family with sequence similarity 53 member B | -2.344 | 4.559 |
| UBIAD1 | UbiA prenyltransferase domain containing 1 | -2.406 | 2.580 |
| FBXL18 | F-box and leucine rich repeat protein 18 | -2.482 | 3.875 |
| CASTOR2 | cytosolic arginine sensor for mTORC1 subunit 2 | -2.572 | 3.705 |
| DOCK3 | dedicator of cytokinesis 3 | -2.635 | 1.945 |
| FGD3 | FYVE, RhoGEF and PH domain containing 3 | -2.689 | 4.471 |
| JRK | Jrk helix-turn-helix protein | -2.815 | 2.178 |
| NMRAL2P | NmrA like redox sensor 2, pseudogene | -3.599 | 3.427 |

**Supplementary Table 2: Significant DEGs (|log2FC|≥1, *p*≤0.05), E24377A infection relative to uninfected control**

| **Gene symbol** | **Gene name** | **log2FC** | **-log10(pval)** |
| --- | --- | --- | --- |
| C2CD4A | C2 calcium dependent domain containing 4A | 4.246 | 2.383 |
| HILPDA | hypoxia inducible lipid droplet associated | 3.579 | 3.861 |
| FEZ1 | fasciculation and elongation protein zeta 1 | 3.507 | 1.760 |
| SLC2A3 | solute carrier family 2 member 3 | 3.453 | 3.603 |
| IL20 | interleukin 20 | 2.695 | 1.316 |
| TUBB2B | tubulin beta 2B class IIb | 2.515 | 1.708 |
| CXCR4 | C-X-C motif chemokine receptor 4 | 2.406 | 14.759 |
| HMGB2 | high mobility group box 2 | 2.398 | 5.664 |
| CSF2 | colony stimulating factor 2 | 2.130 | 2.986 |
| ZNF442 | zinc finger protein 442 | 2.128 | 1.501 |
| G0S2 | G0/G1 switch 2 | 2.084 | 3.736 |
| CXCL2 | C-X-C motif chemokine ligand 2 | 2.072 | 1.894 |
| DDIT4 | DNA damage inducible transcript 4 | 1.926 | 5.218 |
| IL1A | interleukin 1 alpha | 1.870 | 1.310 |
| UNC5B-AS1 | UNC5B antisense RNA 1 | 1.809 | 1.807 |
| NEURL3 | neuralized E3 ubiquitin protein ligase 3 | 1.804 | 1.812 |
| NAB2 | NGFI-A binding protein 2 | 1.740 | 3.305 |
| FOS | Fos proto-oncogene, AP-1 transcription factor subunit | 1.676 | 3.032 |
| ENO2 | enolase 2 | 1.672 | 3.373 |
| GDF15 | growth differentiation factor 15 | 1.650 | 1.774 |
| CCSAP | centriole, cilia and spindle associated protein | 1.640 | 2.176 |
| EDN2 | endothelin 2 | 1.571 | 1.554 |
| PIM1 | Pim-1 proto-oncogene, serine/threonine kinase | 1.558 | 5.291 |
| CX3CL1 | C-X3-C motif chemokine ligand 1 | 1.548 | 1.387 |
| RHOV | ras homolog family member V | 1.519 | 1.450 |
| VEGFA | vascular endothelial growth factor A | 1.514 | 4.246 |
| HLA-L | major histocompatibility complex, class I, L (pseudogene) | 1.496 | 1.377 |
| ADM | adrenomedullin | 1.492 | 2.919 |
| ANKRD37 | ankyrin repeat domain 37 | 1.447 | 5.394 |
| ZNF563 | zinc finger protein 563 | 1.418 | 1.382 |
| EFNA3 | ephrin A3 | 1.409 | 2.783 |
| BBC3 | BCL2 binding component 3 | 1.406 | 2.144 |
| N4BP3 | NEDD4 binding protein 3 | 1.403 | 1.762 |
| CBARP | CACN subunit beta associated regulatory protein | 1.397 | 1.933 |
| CDKN2C | cyclin dependent kinase inhibitor 2C | 1.392 | 1.582 |
| FOXA3 | forkhead box A3 | 1.381 | 3.566 |
| VPS37D | VPS37D subunit of ESCRT-I | 1.311 | 1.793 |
| CRABP2 | cellular retinoic acid binding protein 2 | 1.309 | 3.597 |
| GEM | GTP binding protein overexpressed in skeletal muscle | 1.295 | 2.709 |
| EFNA1 | ephrin A1 | 1.272 | 1.619 |
| DUSP10 | dual specificity phosphatase 10 | 1.267 | 2.205 |
| FRS3 | fibroblast growth factor receptor substrate 3 | 1.262 | 2.967 |
| FBXO32 | F-box protein 32 | 1.246 | 2.676 |
| RSRP1 | arginine and serine rich protein 1 | 1.225 | 2.593 |
| PIM3 | Pim-3 proto-oncogene, serine/threonine kinase | 1.219 | 3.376 |
| N4BP2L1 | NEDD4 binding protein 2 like 1 | 1.185 | 1.383 |
| SLC2A1 | solute carrier family 2 member 1 | 1.161 | 2.714 |
| CDKN2D | cyclin dependent kinase inhibitor 2D | 1.157 | 3.085 |
| RORA | RAR related orphan receptor A | 1.155 | 1.355 |
| NFKBIA | NFKB inhibitor alpha | 1.119 | 1.924 |
| ZFP36 | ZFP36 ring finger protein | 1.079 | 1.367 |
| BHLHE40 | basic helix-loop-helix family member e40 | 1.076 | 2.842 |
| ANGPTL4 | angiopoietin like 4 | 1.066 | 2.310 |
| RELT | RELT TNF receptor | 1.055 | 2.028 |
| RAB3A | RAB3A, member RAS oncogene family | 1.047 | 1.328 |
| ZC3H12A | zinc finger CCCH-type containing 12A | 1.045 | 1.501 |
| BNIP5 | BCL2 interacting protein 5 | 1.025 | 1.412 |
| TRIM16L | tripartite motif containing 16 like (pseudogene) | -1.001 | 3.121 |
| DDX20 | DEAD-box helicase 20 | -1.001 | 1.939 |
| ARAP3 | ArfGAP with RhoGAP domain, ankyrin repeat and PH domain 3 | -1.003 | 2.136 |
| TRIB1 | tribbles pseudokinase 1 | -1.005 | 1.587 |
| FBXO41 | F-box protein 41 | -1.009 | 1.411 |
| HSPA8 | heat shock protein family A (Hsp70) member 8 | -1.011 | 4.833 |
| POLR1B | RNA polymerase I subunit B | -1.014 | 1.637 |
| ADGRL1 | adhesion G protein-coupled receptor L1 | -1.022 | 2.763 |
| PKD1 | polycystin 1, transient receptor potential channel interacting | -1.022 | 2.019 |
| ZCCHC14 | zinc finger CCHC-type containing 14 | -1.024 | 4.971 |
| C1orf226 | chromosome 1 open reading frame 226 | -1.026 | 2.002 |
| TOE1 | target of EGR1, exonuclease | -1.029 | 2.666 |
| PLXNA1 | plexin A1 | -1.031 | 2.665 |
| LTO1 | LTO1 maturation factor of ABCE1 | -1.032 | 3.204 |
| CABLES1 | Cdk5 and Abl enzyme substrate 1 | -1.033 | 2.138 |
| ZBTB40 | zinc finger and BTB domain containing 40 | -1.042 | 5.191 |
| MAVS | mitochondrial antiviral signaling protein | -1.043 | 2.605 |
| FCGBP | Fc gamma binding protein | -1.045 | 1.451 |
| UNKL | unk like zinc finger | -1.048 | 3.309 |
| TRIM35 | tripartite motif containing 35 | -1.050 | 3.380 |
| NR2F2 | nuclear receptor subfamily 2 group F member 2 | -1.052 | 1.755 |
| PUS3 | pseudouridine synthase 3 | -1.056 | 2.009 |
| BAMBI | BMP and activin membrane bound inhibitor | -1.062 | 1.773 |
| CDC42EP3 | CDC42 effector protein 3 | -1.075 | 1.413 |
| NBPF14 | NBPF member 14 | -1.076 | 1.972 |
| SMCR8 | SMCR8-C9orf72 complex subunit | -1.080 | 1.365 |
| LOC729218 | uncharacterized LOC729218 | -1.082 | 1.373 |
| MDN1 | midasin AAA ATPase 1 | -1.084 | 5.402 |
| EPC1 | enhancer of polycomb homolog 1 | -1.084 | 6.378 |
| TRPV1 | transient receptor potential cation channel subfamily V member 1 | -1.085 | 1.476 |
| CENPO | centromere protein O | -1.085 | 2.274 |
| ASTE1 | asteroid homolog 1 | -1.085 | 2.923 |
| RBBP6 | RB binding protein 6, ubiquitin ligase | -1.086 | 3.606 |
| URB1 | URB1 ribosome biogenesis homolog | -1.088 | 1.731 |
| GFOD1 | Gfo/Idh/MocA-like oxidoreductase domain containing 1 | -1.090 | 1.368 |
| NBPF10 | NBPF member 10 | -1.093 | 1.963 |
| CRAMP1 | cramped chromatin regulator homolog 1 | -1.094 | 4.524 |
| TEF | TEF transcription factor, PAR bZIP family member | -1.101 | 1.390 |
| NDST1 | N-deacetylase and N-sulfotransferase 1 | -1.116 | 3.256 |
| ZNF37A | zinc finger protein 37A | -1.118 | 2.795 |
| PSMG3-AS1 | PSMG3 antisense RNA 1 (head to head) | -1.119 | 1.365 |
| GPAM | glycerol-3-phosphate acyltransferase, mitochondrial | -1.122 | 2.568 |
| ZNF557 | zinc finger protein 557 | -1.123 | 2.414 |
| PCNX3 | pecanex 3 | -1.126 | 2.407 |
| XKR8 | XK related 8 | -1.127 | 1.334 |
| ZNF225 | zinc finger protein 225 | -1.127 | 1.684 |
| ZC3H8 | zinc finger CCCH-type containing 8 | -1.130 | 1.909 |
| ARRDC4 | arrestin domain containing 4 | -1.130 | 2.136 |
| ING3 | inhibitor of growth family member 3 | -1.131 | 3.977 |
| TP53INP1 | tumor protein p53 inducible nuclear protein 1 | -1.133 | 2.043 |
| NXPE3 | neurexophilin and PC-esterase domain family member 3 | -1.135 | 2.617 |
| ZNF740 | zinc finger protein 740 | -1.145 | 3.005 |
| TBC1D13 | TBC1 domain family member 13 | -1.147 | 2.436 |
| PLK2 | polo like kinase 2 | -1.149 | 5.415 |
| ZBTB47 | zinc finger and BTB domain containing 47 | -1.156 | 1.388 |
| SLC35E2A | solute carrier family 35 member E2A (pseudogene) | -1.162 | 1.505 |
| AP5S1 | adaptor related protein complex 5 subunit sigma 1 | -1.172 | 4.151 |
| ACACB | acetyl-CoA carboxylase beta | -1.174 | 3.118 |
| MEF2C | myocyte enhancer factor 2C | -1.179 | 2.082 |
| USP22 | ubiquitin specific peptidase 22 | -1.183 | 2.681 |
| PIK3CD | phosphatidylinositol-4,5-bisphosphate 3-kinase catalytic subunit delta | -1.188 | 1.635 |
| IP6K1 | inositol hexakisphosphate kinase 1 | -1.190 | 2.807 |
| KDM8 | lysine demethylase 8 | -1.197 | 3.305 |
| DGKD | diacylglycerol kinase delta | -1.198 | 2.411 |
| ZC3HAV1 | zinc finger CCCH-type containing, antiviral 1 | -1.215 | 3.696 |
| CCDC85C | coiled-coil domain containing 85C | -1.218 | 2.784 |
| NUAK1 | NUAK family kinase 1 | -1.232 | 2.078 |
| NPY4R2 | neuropeptide Y receptor Y4-2 | -1.242 | 1.584 |
| SLC7A11 | solute carrier family 7 member 11 | -1.245 | 1.467 |
| SUV39H1 | SUV39H1 histone lysine methyltransferase | -1.247 | 2.048 |
| LCAT | lecithin-cholesterol acyltransferase | -1.250 | 1.395 |
| MEGF8 | multiple EGF like domains 8 | -1.252 | 2.494 |
| SRXN1 | sulfiredoxin 1 | -1.254 | 1.604 |
| B3GALT5 | beta-1,3-galactosyltransferase 5 | -1.254 | 3.146 |
| ZNF552 | zinc finger protein 552 | -1.262 | 2.607 |
| PLCXD2 | phosphatidylinositol specific phospholipase C X domain containing 2 | -1.263 | 2.630 |
| NBPF12 | NBPF member 12 | -1.265 | 7.187 |
| SGK1 | serum/glucocorticoid regulated kinase 1 | -1.266 | 3.953 |
| POU2AF3 | POU class 2 homeobox associating factor 3 | -1.268 | 1.483 |
| IRGQ | immunity related GTPase Q | -1.272 | 1.829 |
| E2F6 | E2F transcription factor 6 | -1.273 | 2.923 |
| NACC2 | NACC family member 2 | -1.273 | 4.423 |
| MEPCE | methylphosphate capping enzyme | -1.273 | 2.187 |
| HIRA | histone cell cycle regulator | -1.282 | 3.147 |
| ANKRD52 | ankyrin repeat domain 52 | -1.285 | 2.583 |
| TBC1D16 | TBC1 domain family member 16 | -1.287 | 4.447 |
| IFFO2 | intermediate filament family orphan 2 | -1.290 | 3.290 |
| HDAC4 | histone deacetylase 4 | -1.294 | 4.635 |
| PCF11 | PCF11 cleavage and polyadenylation factor subunit | -1.299 | 5.225 |
| ZNF180 | zinc finger protein 180 | -1.302 | 3.447 |
| CMTM4 | CKLF like MARVEL transmembrane domain containing 4 | -1.321 | 1.859 |
| XYLT1 | xylosyltransferase 1 | -1.325 | 3.440 |
| ATXN7L2 | ataxin 7 like 2 | -1.340 | 3.388 |
| TGM1 | transglutaminase 1 | -1.342 | 1.503 |
| URB2 | URB2 ribosome biogenesis homolog | -1.369 | 2.355 |
| NBPF1 | NBPF member 1 | -1.385 | 1.889 |
| ADSS1 | adenylosuccinate synthase 1 | -1.391 | 3.625 |
| MAML1 | mastermind like transcriptional coactivator 1 | -1.394 | 2.066 |
| FOXK1 | forkhead box K1 | -1.394 | 1.505 |
| WDR37 | WD repeat domain 37 | -1.403 | 6.281 |
| WDR4 | WD repeat domain 4 | -1.405 | 1.639 |
| PKD1P1 | polycystin 1, transient receptor potential channel interacting pseudogene 1 | -1.421 | 1.389 |
| NWD1 | NACHT and WD repeat domain containing 1 | -1.429 | 2.331 |
| VCPKMT | valosin containing protein lysine methyltransferase | -1.436 | 2.213 |
| FDXACB1 | ferredoxin-fold anticodon binding domain containing 1 | -1.454 | 1.480 |
| FAM168B | family with sequence similarity 168 member B | -1.456 | 1.883 |
| OVOL2 | ovo like zinc finger 2 | -1.457 | 1.794 |
| CEL | carboxyl ester lipase | -1.467 | 1.455 |
| GPR37L1 | G protein-coupled receptor 37 like 1 | -1.487 | 1.503 |
| GRAMD1B | GRAM domain containing 1B | -1.488 | 3.402 |
| TMEM140 | transmembrane protein 140 | -1.491 | 2.101 |
| RTL6 | retrotransposon Gag like 6 | -1.494 | 1.440 |
| EFR3B | EFR3 homolog B | -1.500 | 1.802 |
| ZNF35 | zinc finger protein 35 | -1.501 | 2.361 |
| DANCR | differentiation antagonizing non-protein coding RNA | -1.504 | 1.445 |
| NCOA5 | nuclear receptor coactivator 5 | -1.518 | 8.701 |
| PRDM11 | PR/SET domain 11 | -1.526 | 2.555 |
| ZNF324B | zinc finger protein 324B | -1.537 | 1.392 |
| AVIL | advillin | -1.537 | 2.218 |
| ZNF416 | zinc finger protein 416 | -1.542 | 1.572 |
| HR | HR lysine demethylase and nuclear receptor corepressor | -1.548 | 2.687 |
| TRIM25 | tripartite motif containing 25 | -1.563 | 3.603 |
| DTX4 | deltex E3 ubiquitin ligase 4 | -1.596 | 3.802 |
| PSKH1 | protein serine kinase H1 | -1.601 | 2.085 |
| FBXL18 | F-box and leucine rich repeat protein 18 | -1.616 | 1.947 |
| ZNF689 | zinc finger protein 689 | -1.626 | 4.095 |
| CAD | carbamoyl-phosphate synthetase 2, aspartate transcarbamylase, and dihydroorotase | -1.640 | 2.801 |
| ENC1 | ectodermal-neural cortex 1 | -1.663 | 1.910 |
| UQCC4 | ubiquinol-cytochrome c reductase complex assembly factor 4 | -1.671 | 2.501 |
| LOC105376526 | uncharacterized LOC105376526 | -1.679 | 1.389 |
| HAPLN4 | hyaluronan and proteoglycan link protein 4 | -1.710 | 1.372 |
| IBA57 | iron-sulfur cluster assembly factor IBA57 | -1.712 | 1.885 |
| ARHGEF37 | Rho guanine nucleotide exchange factor 37 | -1.736 | 4.424 |
| ZNF319 | zinc finger protein 319 | -1.749 | 2.803 |
| LINC01269 | long intergenic non-protein coding RNA 1269 | -1.806 | 3.243 |
| CCNA2 | cyclin A2 | -1.807 | 1.783 |
| WDR62 | WD repeat domain 62 | -1.819 | 1.451 |
| ZNF696 | zinc finger protein 696 | -1.858 | 1.892 |
| BEST3 | bestrophin 3 | -1.886 | 1.516 |
| FAM13B | family with sequence similarity 13 member B | -1.893 | 8.735 |
| TWNK | twinkle mtDNA helicase | -1.925 | 1.785 |
| MSL2 | MSL complex subunit 2 | -1.966 | 2.222 |
| UBIAD1 | UbiA prenyltransferase domain containing 1 | -1.974 | 1.946 |
| CYTIP | cytohesin 1 interacting protein | -1.990 | 1.351 |
| CYP26B1 | cytochrome P450 family 26 subfamily B member 1 | -2.167 | 2.155 |
| MGAT3 | beta-1,4-mannosyl-glycoprotein 4-beta-N-acetylglucosaminyltransferase | -2.207 | 2.999 |
| UNC119B | unc-119 lipid binding chaperone B | -2.249 | 4.735 |
| CASTOR2 | cytosolic arginine sensor for mTORC1 subunit 2 | -2.380 | 3.244 |
| FAM53B | family with sequence similarity 53 member B | -2.484 | 4.979 |
| CYB561D1 | cytochrome b561 family member D1 | -2.625 | 5.650 |
| ZNF773 | zinc finger protein 773 | -2.719 | 2.960 |
| EGR1 | early growth response 1 | -2.818 | 1.777 |
| PFKFB1 | 6-phosphofructo-2-kinase/fructose-2,6-biphosphatase 1 | -2.853 | 2.579 |
| NMRAL2P | NmrA like redox sensor 2, pseudogene | -3.701 | 3.545 |

**Supplemental Table 3. Shared and distinct DEGs induced by H10407 or E24377A infection.**

| **Shared** | **Unique to H10407** | **Unique to E24377A** |
| --- | --- | --- |
| C2CD4A | OLR1 | ZNF442 |
| HILPDA | TNFAIP6 | UNC5B-AS1 |
| FEZ1 | KIT | ZNF563 |
| SLC2A3 | CCL8 | CBARP |
| IL20 | IL17C | SLC2A1 |
| TUBB2B | NOD2 | DDX20 |
| CXCR4 | PGLYRP2 | ADGRL1 |
| HMGB2 | OVOS2 | ZCCHC14 |
| CSF2 | PTP4A3 | PLXNA1 |
| G0S2 | BCL2A1 | ZBTB40 |
| CXCL2 | IL19 | FCGBP |
| DDIT4 | GFPT2 | UNKL |
| IL1A | SNPH | TRIM35 |
| NEURL3 | NOS2 | NR2F2 |
| NAB2 | WFDC5 | PUS3 |
| FOS | CD83 | NBPF14 |
| ENO2 | PLA2G4E | SMCR8 |
| GDF15 | KRT6B | LOC729218 |
| CCSAP | SLFN11 | TRPV1 |
| EDN2 | NKX3-1 | RBBP6 |
| PIM1 | CXCL6 | GFOD1 |
| CX3CL1 | NPHS1 | NBPF10 |
| RHOV | SLC43A3 | CRAMP1 |
| VEGFA | CCL5 | TEF |
| HLA-L | IL1B | ZNF37A |
| ADM | SLC6A12 | GPAM |
| ANKRD37 | CCL20 | ZNF557 |
| EFNA3 | TIFA | XKR8 |
| BBC3 | SLC5A5 | ARRDC4 |
| N4BP3 | CCL7 | NXPE3 |
| CDKN2C | SELE | ZNF740 |
| FOXA3 | ZNF385C | ZBTB47 |
| VPS37D | FMNL3 | SLC35E2A |
| CRABP2 | ARHGAP31 | AP5S1 |
| GEM | C2CD4B | IP6K1 |
| EFNA1 | CLEC2D | ZC3HAV1 |
| DUSP10 | DOC2B | CCDC85C |
| FRS3 | REM2 | NPY4R2 |
| FBXO32 | ICAM1 | ZNF552 |
| RSRP1 | SOD2 | SGK1 |
| PIM3 | TMOD2 | POU2AF3 |
| N4BP2L1 | UBASH3A | IRGQ |
| CDKN2D | CCL22 | E2F6 |
| RORA | RHCG | TGM1 |
| NFKBIA | EBI3 | MAML1 |
| ZFP36 | CXCL1 | FOXK1 |
| BHLHE40 | UNC13A | PKD1P1 |
| ANGPTL4 | KIRREL2 | VCPKMT |
| RELT | NPR1 | OVOL2 |
| RAB3A | NCCRP1 | GPR37L1 |
| ZC3H12A | TNFRSF9 | TMEM140 |
| BNIP5 | PTGS2 | RTL6 |
| TRIM16L | LOC102723996 | ZNF324B |
| ARAP3 | CBFA2T3 | LOC105376526 |
| TRIB1 | ICAM5 | HAPLN4 |
| FBXO41 | CARD14 | IBA57 |
| HSPA8 | SLC6A14 | CCNA2 |
| POLR1B | FERMT3 | WDR62 |
| PKD1 | SEMA3B-AS1 | BEST3 |
| C1orf226 | ITPRID1 | MSL2 |
| TOE1 | RND1 | CYTIP |
| LTO1 | CHST2 | PFKFB1 |
| CABLES1 | IL18R1 |  |
| MAVS | IL10RA |  |
| BAMBI | NR4A1 |  |
| CDC42EP3 | CPNE5 |  |
| MDN1 | BPGM |  |
| EPC1 | DLL4 |  |
| CENPO | SDC4 |  |
| ASTE1 | JCAD |  |
| URB1 | CXCL5 |  |
| NDST1 | SLCO4A1 |  |
| PSMG3-AS1 | ADGRB2 |  |
| PCNX3 | CXCL10 |  |
| ZNF225 | GNA15 |  |
| ZC3H8 | CD69 |  |
| ING3 | LOC105371730 |  |
| TP53INP1 | LINC00957 |  |
| TBC1D13 | ENKUR |  |
| PLK2 | RASGEF1A |  |
| ACACB | TNFAIP3 |  |
| MEF2C | CXCL3 |  |
| USP22 | BEAN1 |  |
| PIK3CD | SH2D1B |  |
| KDM8 | MAP3K8 |  |
| DGKD | TRAF1 |  |
| NUAK1 | PRKD3 |  |
| SLC7A11 | CEP112 |  |
| SUV39H1 | C3 |  |
| LCAT | DENND2B |  |
| MEGF8 | NFATC1 |  |
| SRXN1 | ICOSLG |  |
| B3GALT5 | CXCL8 |  |
| PLCXD2 | XDH |  |
| NBPF12 | PRDM1 |  |
| NACC2 | DNAH17 |  |
| MEPCE | C10orf55 |  |
| HIRA | PLAT |  |
| ANKRD52 | PSTPIP2 |  |
| TBC1D16 | PTCH1 |  |
| IFFO2 | TNFSF9 |  |
| HDAC4 | PI3 |  |
| PCF11 | SERPINB7 |  |
| ZNF180 | BIRC3 |  |
| CMTM4 | RASGRP1 |  |
| XYLT1 | IL23A |  |
| ATXN7L2 | NAMPT |  |
| URB2 | STAT5A |  |
| NBPF1 | TNFAIP2 |  |
| ADSS1 | WNT4 |  |
| WDR37 | BDKRB1 |  |
| WDR4 | SOX7 |  |
| NWD1 | CD44 |  |
| FDXACB1 | CSF1 |  |
| FAM168B | CHAC1 |  |
| CEL | MMP25-AS1 |  |
| GRAMD1B | NFKB2 |  |
| EFR3B | NIBAN1 |  |
| ZNF35 | CA5B |  |
| DANCR | PLEKHF1 |  |
| NCOA5 | SAT1 |  |
| PRDM11 | WFDC3 |  |
| AVIL | LOC101928596 |  |
| HR | STX11 |  |
| TRIM25 | CHRNA5 |  |
| DTX4 | GAS7 |  |
| PSKH1 | STARD5 |  |
| FBXL18 | NOXO1 |  |
| ZNF689 | BAZ1A |  |
| CAD | NFKBIZ |  |
| ENC1 | RAB24 |  |
| ARHGEF37 | BCOR |  |
| LINC01269 | DUSP5 |  |
| ZNF696 | CISH |  |
| FAM13B | SLC25A22 |  |
| TWNK | MAFF |  |
| UBIAD1 | PFKFB3 |  |
| CYP26B1 | COL16A1 |  |
| MGAT3 | B3GNT2 |  |
| UNC119B | SIK1 |  |
| CASTOR2 | ANKRD36C |  |
| FAM53B | APOBEC3G |  |
| CYB561D1 | GCH1 |  |
| EGR1 | WFDC12 |  |
| NMRAL2P | CCNE2 |  |
|  | PLAU |  |
|  | HIF1A |  |
|  | PRSS53 |  |
|  | TNNT2 |  |
|  | PLEKHA4 |  |
|  | IFT57 |  |
|  | NR4A2 |  |
|  | ISG15 |  |
|  | STEAP4 |  |
|  | CNOT8 |  |
|  | SLC28A3 |  |
|  | SLC25A48 |  |
|  | OGFRL1 |  |
|  | COL9A2 |  |
|  | FAM241A |  |
|  | ADAMTS9 |  |
|  | KHDRBS3 |  |
|  | RAC2 |  |
|  | TRIP10 |  |
|  | DRAM1 |  |
|  | LYSMD2 |  |
|  | RWDD2A |  |
|  | ALCAM |  |
|  | CYLD |  |
|  | NECTIN4 |  |
|  | EIF4A3 |  |
|  | EFNA4 |  |
|  | ETV7 |  |
|  | ERO1A |  |
|  | NFKBIE |  |
|  | ARRDC1-AS1 |  |
|  | TBC1D10A |  |
|  | CLK3 |  |
|  | BCL2L11 |  |
|  | PHF20 |  |
|  | SERTAD2 |  |
|  | TBC1D2B |  |
|  | HIVEP2 |  |
|  | ACYP1 |  |
|  | PIGA |  |
|  | RAPGEF3 |  |
|  | BATF2 |  |
|  | CLK1 |  |
|  | JAK2 |  |
|  | RHBDF2 |  |
|  | PIM2 |  |
|  | RNF223 |  |
|  | TNFRSF1B |  |
|  | LYN |  |
|  | RALGDS |  |
|  | BTG3 |  |
|  | OAF |  |
|  | TNIP1 |  |
|  | FAM210A |  |
|  | MXD1 |  |
|  | PER1 |  |
|  | AVPI1 |  |
|  | LOC102724428 |  |
|  | DCUN1D3 |  |
|  | SAV1 |  |
|  | CDKN1A |  |
|  | S100A3 |  |
|  | BID |  |
|  | PPP4R2 |  |
|  | ANKRD36 |  |
|  | DNTTIP1 |  |
|  | IL32 |  |
|  | POU2F2 |  |
|  | RSRC2 |  |
|  | ISG20 |  |
|  | SMNDC1 |  |
|  | ANKRD36B |  |
|  | TMPRSS2 |  |
|  | BCL6 |  |
|  | TRERF1 |  |
|  | BARX2 |  |
|  | HK2 |  |
|  | REL |  |
|  | NFKB1 |  |
|  | PSMA6 |  |
|  | CENPW |  |
|  | LNX1 |  |
|  | CSRNP1 |  |
|  | ST3GAL1 |  |
|  | TMEM106A |  |
|  | IKBKE |  |
|  | TMEM74B |  |
|  | RILPL2 |  |
|  | PTMS |  |
|  | SERTAD3 |  |
|  | TIPARP |  |
|  | ZHX2 |  |
|  | ING1 |  |
|  | ATP6V1C2 |  |
|  | BTN2A3P |  |
|  | PLAUR |  |
|  | SLC2A6 |  |
|  | DPM3 |  |
|  | FAM219A |  |
|  | NUB1 |  |
|  | SLC25A25 |  |
|  | GCKR |  |
|  | TNFSF10 |  |
|  | ZNF622 |  |
|  | RELB |  |
|  | PELI1 |  |
|  | RAP1GAP |  |
|  | MMP10 |  |
|  | CCNG1 |  |
|  | MRTO4 |  |
|  | KRT80 |  |
|  | TCEANC |  |
|  | ZNF678 |  |
|  | SFT2D2 |  |
|  | METTL1 |  |
|  | MTCL2 |  |
|  | HGH1 |  |
|  | PHETA2 |  |
|  | NBPF26 |  |
|  | IQCE |  |
|  | TNS3 |  |
|  | CEP78 |  |
|  | SLC35B4 |  |
|  | ZBED4 |  |
|  | CCNJ |  |
|  | USP46 |  |
|  | TNRC6B |  |
|  | FOXA1 |  |
|  | NR2C1 |  |
|  | GTF2E1 |  |
|  | LAMA5 |  |
|  | BBS5 |  |
|  | CASC19 |  |
|  | CASTOR3P |  |
|  | KANSL3 |  |
|  | ARHGAP29 |  |
|  | TACC3 |  |
|  | SBF1 |  |
|  | GNAL |  |
|  | FBXO9 |  |
|  | NOD1 |  |
|  | ARL4A |  |
|  | DEPDC7 |  |
|  | BYSL |  |
|  | DCAF4 |  |
|  | ZXDC |  |
|  | ZNF202 |  |
|  | ZNF362 |  |
|  | CCDC171 |  |
|  | EXTL3 |  |
|  | SARM1 |  |
|  | PTPRU |  |
|  | CDCA7L |  |
|  | NF2 |  |
|  | NBEAL2 |  |
|  | PITPNM3 |  |
|  | MICAL2 |  |
|  | ZNF605 |  |
|  | CDON |  |
|  | CCDC142 |  |
|  | MYOM3 |  |
|  | C21orf91 |  |
|  | ISX |  |
|  | DDX31 |  |
|  | ZNF75A |  |
|  | ZSCAN20 |  |
|  | CEP76 |  |
|  | ANKS6 |  |
|  | RBM12B |  |
|  | TLN2 |  |
|  | NT5DC2 |  |
|  | DHX35 |  |
|  | FAM217B |  |
|  | CLDN10-AS1 |  |
|  | PFAS |  |
|  | POLR3H |  |
|  | CDR2 |  |
|  | TRMT5 |  |
|  | MFNG |  |
|  | ZNF786 |  |
|  | GEN1 |  |
|  | RAB3D |  |
|  | WARS2-AS1 |  |
|  | KNOP1 |  |
|  | KITLG |  |
|  | PHETA1 |  |
|  | ZNF8 |  |
|  | VANGL1 |  |
|  | NCS1 |  |
|  | RBM4B |  |
|  | NHSL1 |  |
|  | PLEKHA8P1 |  |
|  | POLR1A |  |
|  | SLC29A3 |  |
|  | LMO4 |  |
|  | CELSR1 |  |
|  | MTARC1 |  |
|  | STON2 |  |
|  | CBX6 |  |
|  | SRGAP1 |  |
|  | TSEN2 |  |
|  | ZNF302 |  |
|  | NIPAL1 |  |
|  | ADCY7 |  |
|  | GCLC |  |
|  | BICD2 |  |
|  | LRRC20 |  |
|  | SPRY2 |  |
|  | C11orf86 |  |
|  | MMP24 |  |
|  | RARB |  |
|  | COQ10A |  |
|  | EXO5 |  |
|  | SPTBN2 |  |
|  | CENPL |  |
|  | MTERF1 |  |
|  | ZBTB3 |  |
|  | XRCC3 |  |
|  | L3HYPDH |  |
|  | SORCS2 |  |
|  | TNS4 |  |
|  | ZNF772 |  |
|  | OSGIN1 |  |
|  | LRRC66 |  |
|  | DOK7 |  |
|  | KRT10 |  |
|  | UGT1A6 |  |
|  | ZNF888 |  |
|  | TRAM2 |  |
|  | LINC00857 |  |
|  | HPX |  |
|  | MLXIP |  |
|  | MAT2A |  |
|  | THBS1 |  |
|  | CYTH3 |  |
|  | EFNB2 |  |
|  | KIF23 |  |
|  | CCDC113 |  |
|  | BCDIN3D |  |
|  | MCM3AP-AS1 |  |
|  | PPARGC1B |  |
|  | LMCD1 |  |
|  | ADAMTS15 |  |
|  | TMEM131L |  |
|  | KAT14 |  |
|  | EPHA4 |  |
|  | ADGRF5 |  |
|  | PCED1B |  |
|  | PIH1D2 |  |
|  | SPATA33 |  |
|  | HMGA2 |  |
|  | ITIH4 |  |
|  | COMETT |  |
|  | CELSR3 |  |
|  | TXK |  |
|  | RGS2 |  |
|  | MITF |  |
|  | RGS5 |  |
|  | HCN4 |  |
|  | ZSCAN31 |  |
|  | BAZ2B-AS1 |  |
|  | FRMPD1 |  |
|  | SLC22A20P |  |
|  | LINC01588 |  |
|  | CCAT1 |  |
|  | DTNA |  |
|  | PHACTR3 |  |
|  | ANKRD44 |  |
|  | PDE7B |  |
|  | HS3ST5 |  |
|  | DOCK3 |  |
|  | FGD3 |  |
|  | JRK |  |

**Supplementary Table 4: Bacterial strains and plasmids**

| **Bacterial Strain** | **Description** | **Reference** |
| --- | --- | --- |
| Enterotoxigenic *E. coli* B7A | serotype O148:H28; LT+, STh+, CS6 | (1) |
| Enterotoxigenic *E. coli* E24377A | serotype O139:H28; LT+, STh+, STp+, EtpA+, CS1, CS3 | (2) |
| Enterotoxigenic *E. coli* H10407 | serotype O78:H11; LT+, STh+, STp+, EtpA+, CFA/I | (3) |
| Enterotoxigenic *E. coli* H10407 ΔfliC::Km^R^ | jf1412; LT+, STh+, STp+, EtpA+, CFA/I, *fliC*::*kanR* | (4) |
| Enterotoxigenic E. coli H10407 WT:pJFA014 | jfa007; inducible expression of functional *fliZ* copy | This study |
| Enterotoxigenic E. coli H10407 WT:pTrcHis A | jfa006; empty plasmid control | This study |
| *E. coli* HS | serotype O9:H4; commensal isolated from a healthy lab technician |  |
| *E. coli* HS WT:pJFA007 | jfa010; constitutive expression mScarlet-I reporter; AmpR | This study |
| *E. coli* TOP10 | K-12 derivative; flagella-deficient | Invitrogen |
| **Plasmid** | **Description** | **Reference** |
| pTrcHis A | IPTG-inducible plasmid for N-terminal 6xHis fusion expression; AmpR | Invitrogen |
| 23ABFURD | custom Invitrogen GeneArt plasmid containing FliC-H4 flanked by BamHI and HindIII sites; KanR | This study |
| 23ABFUTD | custom Invitrogen GeneArt plasmid containing FliC-H11 flanked by BamHI and HindIII sites; KanR | This study |
| 23ABFUUD | custom Invitrogen GeneArt plasmid containing FliC-H28 flanked by BamHI and HindIII sites; KanR | This study |
| pJFA004 | serotype H4 FliC expression plasmid; FliC-H4 excised from plasmid 23ABFURD and cloned into pTrcHis A at BamHI and HindIII sites; AmpR | This study |
| pJFA005 | serotype H11 FliC expression plasmid; FliC-H11 excised from plasmid 23ABFUTD and cloned into pTrcHis A at BamHI and HindIII sites; AmpR | This study |
| pJFA006 | serotype H28 FliC expression plasmid; FliC-H28 excised from plasmid 23ABFUUD and cloned into pTrcHis A at BamHI and HindIII sites; AmpR | This study |
| pJFA007 | mScarlet-I reporter cloned into KpnI and HindIII sites of pUC19 | This study |
| pJFA014 | fliZ amplified from *E. coli* K-12 using primers fliZ-BamHI-corr F and fliZ-EcoRI F; cloned in BamHI and EcoRI sites of pTrcHis A for N-terminal 6xHis fusion expression; AmpR | This study |

**Supplementary Table 5: Primer and shRNA sequences**

| **Cloning primer** | **Sequence (5'-3')** |
| --- | --- |
| fliZ-BamHI-corr F | TATAGGATCCATGATGGTGCAGCACCTG |
| fliZ-EcoRI R | TGGTGAATTCTCAATATATATCAGAAGAAGGCAGGCTGGAGG |
| mScarlet-KpnI F | AAGGTACCATGGTGAGCAAGGGCGAGGCA |
| mScarlet-HindIII R | TCAAGCTTACTTGTACAGCTCGTCCATGCC |
| **EMSA probe primer** | **Sequence (5'-3')** |
| flhDCp-IRDye700 F | IRDye700-CGTGTAGTGACGAGTACAGTTGC |
| flhDCp-IRDye700 R | IRDye700-GTTTCAGCAACTCGGAGGTATG |
| **qPCR primer** | **Sequence (5' - 3')** |
| CCL8 F | AACCTTCACCTCTCATGCTG |
| CCL8 R | TGGAAACTGAATCTGGCTGAG |
| CD83 F | AAATACAGAGCGGAGATTGTCC |
| CD83 R | GGAGGTAACTGGGAGAAAAGC |
| CXCL6 F | GTTTGTCTGGACCCGGAAG |
| CXCL6 R | GTCCAGGGATCTCCAGAAAAC |
| IL17C F | GAGGTGTTGGAGGCAGAC |
| IL17C R | CAGCTTCTGTGGATAGCGG |
| IL6 F | CCACTCACCTCTTCAGAACG |
| IL6 R | CATCTTTGGAAGGTTCAGGTTG |
| OLR1 F | GTTGAAGTTCGTGACTGCTTC |
| OLR1 R | TGGAGAGTAAAGAAACTGAAGACC |
| PGLYRP2 F | GCCAACCAGAAATACCCCTT |
| PGLYRP2 R | GATGACAGAGTCCATGAGCAG |
| TLR5 shRNA KD F | GCTAGGACAACGAGGATCATG |
| TLR5 shRNA KD R | GAGGTTGCAGAAACGATAAAAGG |
| **shRNA** | **Mature antisense sequence (5’-3’)** |
| TLR5_V3LHS_332031 | TTTTGGATAGATCCAAGCG |
| TLR5_V3LHS_332026 | TAAATTTCTGAAATAACCA |
| TLR5_V3LHS_332029 | TGAGTGTCTCAAAGACTCG |

**Supplementary Table 6: Organoid and PBMC donor characteristics**

| **Jejunal Organoids** | | |
| --- | --- | --- |
| **Donor ID** | **Sex** | **Age** |
| 1 | F | n.r. |
| 2 | F | n.r. |
| 3 | F | 46 |
| 4 | F | 72 |
| 5 | M | 64 |
| 6 | M | 38 |
| **PBMCs** | | |
| **Donor ID** | **Sex** | **Age** |
| 3906 | M | 28 |
| 5725 | M | 31 |
| 6705 | F | 47 |
| 7396 | F | 53 |
| 9215 | M | 33 |

n.r., not recorded

**Supplementary References**

1.	Rasko DA, Rosovitz MJ, Myers GS, Mongodin EF, Fricke WF, Gajer P, Crabtree J, Sebaihia M, Thomson NR, Chaudhuri R, Henderson IR, Sperandio V, Ravel J. 2008. The pangenome structure of Escherichia coli: comparative genomic analysis of E. coli commensal and pathogenic isolates. J Bacteriol 190:6881-93.

2.	Sahl JW, Rasko DA. 2012. Analysis of global transcriptional profiles of enterotoxigenic Escherichia coli isolate E24377A. Infect Immun 80:1232-42.

3.	Evans DJ, Jr., Evans DG. 1973. Three characteristics associated with enterotoxigenic Escherichia coli isolated from man. Infect Immun 8:322-8.

4.	Roy K, Hilliard GM, Hamilton DJ, Luo J, Ostmann MM, Fleckenstein JM. 2009. Enterotoxigenic Escherichia coli EtpA mediates adhesion between flagella and host cells. Nature 457:594-8.
